# Nuclear complement C3b promotes paclitaxel resistance by assembling the SIN3A/HDAC1/2 complex in non-small cell lung cancer

**DOI:** 10.1038/s41419-023-05869-y

**Published:** 2023-06-08

**Authors:** Xiaochao Wang, Yan Hao, Jianfeng Chen, Peipei Ding, Xinyue Lv, Danlei Zhou, Ling Li, Luying Li, Yanqing Xu, Yumeng Zhu, Wei Zhang, Lu Chen, Tian Liao, Xianghuo He, Qing-Hai Ji, Weiguo Hu

**Affiliations:** 1 Fudan University Shanghai Cancer Center and Institutes of Biomedical Sciences, Shanghai Medical College, Fudan University, Shanghai, 200032 China; 2 State Key Laboratory of Oncology in South China, Collaborative Innovation Center for Cancer Medicine, Sun Yat-sen University Cancer Center, 651 East Dongfeng Road, Guangzhou, Guangdong 510060 China; 3 Department of Head and Neck Surgery, Fudan University Shanghai Cancer Center, Department of Oncology, Shanghai Medical College, Fudan University, Shanghai, 200032 China; 4 Key Laboratory of Breast Cancer in Shanghai, Fudan University Shanghai Cancer Center, Fudan University, Shanghai, 200032 China

**Keywords:** Apoptosis, Mechanisms of disease

## Abstract

In addition to the classical role as a serum effector system of innate immunity, accumulating evidence suggests that intracellular complement components have indispensable functions in immune defense, T cell homeostasis, and tumor cell proliferation and metastasis. Here, we revealed that complement component 3 (C3) is remarkably upregulated in paclitaxel (PTX)-resistant non-small cell lung cancer (NSCLC) cells and that knockdown of C3 promoted PTX-induced cell apoptosis, sensitizing resistant cells to PTX therapy. Ectopic C3 decreased PTX-induced apoptosis and induced resistance to PTX treatment in original NSCLC cells. Interestingly, C3b, the activated fragment of C3, was found to translocate into the nucleus and physically associate with the HDAC1/2-containing SIN3A complex to repress the expression of GADD45A, which plays an important role in cell growth inhibition and apoptosis induction. Importantly, C3 downregulated GADD45A by enhancing the binding of the SIN3A complex with the promoter of GADD45A, thus decreasing the H3Ac level to compress chromatin around the GADD45A locus. Subsequently, ectopic GADD45A promoted PTX-induced cell apoptosis, sensitizing resistant cells to PTX therapy, and insufficiency of GADD45A in original cancer cells induced resistance to PTX treatment. These findings identify a previously unknown nucleus location and oncogenic property for C3 in chemotherapy and provide a potential therapeutic opportunity to overcome PTX resistance.

## Introduction

Chemotherapy is still the most commonly used strategy for treating a wide variety of cancers. In chemotherapy regimens, paclitaxel (PTX) has become one of the most common integrants for treating different types of cancer. It may arrest exposed cancer cells in the G2/M-phase by stabilizing the microtubule polymer, eventually leading to cell apoptosis. Unfortunately, PTX resistance is commonly observed as with other chemotherapeutic agents and is a major obstacle for the success of cancer therapy. Although diverse PTX resistance mechanisms have been reported, including the upregulation of ABC transporters, structural alterations to drug target β-tubulin, and the involvement of cytokines, miRNAs, glucose and lipid metabolism, kinase signaling pathways, and epithelial-mesenchymal transition, the resultant strategies to reverse PTX resistance are still unsatisfactory [1, 2].

Lung cancer is the 2nd most common cancer type and the leading cause of cancer-related deaths worldwide and in the United States [3]. Among all lung cancer cases, non-small cell lung cancer (NSCLC) accounts for 80–85%, and nearly 70% of NSCLC patients present with an advanced stage at the time of diagnosis [4, 5], leaving chemotherapy as the principal treatment strategy, in which PTX is a commonly used drug for late-stage NSCLC patients without benefit from targeted therapy or immunotherapy. However, most patients invariably develop resistance to chemotherapeutic drugs, including PTX, upon prolonged treatment, and the 5-year survival rate remains 20–30% [6, 7]. Therefore, exploring the molecular mechanism of chemoresistance is of great significance to improve the survival rate and quality of life for NSCLC patients.

Complement was originally recognized as a circulating effector system of innate immunity, with hepatocytes producing the majority of soluble complement proteins [8, 9]. Complement activation has long been considered to take place almost exclusively in the blood, resulting in the generation of C3 and C5 convertases, which cleave C3 into C3a and C3b and C5 into C5a and C5b, respectively. C3a and especially C5a induce mobilization of innate immune cells and induction of inflammation; C3b leads to opsonization and removal of invading microbes [10–12], while deposition of C5b onto a target initiates membrane attack complex (MAC) formation and then target lysis. Thus, complement has been assumed to have a beneficial role in the destruction of tumor cells [13]. In contrast to this dogma, it has been reported that C5a generated in the tumor microenvironment may foster tumor initiation and progression by recruiting MDSCs to impair the local T-cell response [14, 15]. In addition, evidence is accumulating that almost all cell types can produce complement components and process complement activation intracellularly, including myeloid, lymphocytic, fibroblastic, epithelial and cancer cells [16, 17]. Moreover, it is now becoming clear that locally activated complement has different functions from systemic serum complement. For example, intracellular C3a is involved in T-cell homeostasis and adaptive immune responses [18, 19]. Autocrine C3 of CD8^+^ T cells may suppress local CD8^+^ T-cell function by inhibiting C3a/C3aR-induced IL-10 production, thus impairing antitumor activity [20]. While B cells, which express little endogenous C3, have been demonstrated to endocytose serum-derived complement C3, the engulfed C3 may enter the nucleus to regulate gene transcription, likely by binding to histones [21]. In addition, tumor cell-derived C3a was reported to promote ovarian cell proliferation via the C3a/C3aR/PI3K/AKT signaling axis [22], EMT of breast cancer cells [23], and leptomeningeal metastasis of lung cancer [24]. In addition, we recently reported a tumorigenic role for intracellular C5a/C5aR1 in colon cancer cells [17]. Cathepsin D cleaves C5 in lysosomes and endosomes, leading to the production of C5a and the assembly of the β-catenin stabilization complex C5a/C5aR1/KCTD5/cullin3/Roc-1, which potentiates β-catenin stability and promotes colorectal tumorigenesis [17]. However, whether intracellular complement components are involved in chemoresistance remains unknown.

Posttranslational modifications (PTMs) of histones, which are the main epigenetic aberrations, have been demonstrated to be involved in tumorigenesis and acquired cancer chemoresistance [25]. The two most common types of modification, acetylation and methylation, occur at either histone H3 or H4 moieties and are orchestrated to regulate gene transcriptional activation or silencing [26, 27]. These PTMs are initiated by histone acetyl transferases and histone methyl transferases. Similarly, these modifications can be reversed by histone deacetylases and histone demethylases [28–30]. SIN3A is a core component of the histone deacetylation activity-associated transcriptional repressor complex, playing an important role in governing cell cycle control and apoptosis throughout tumorigenesis and development [31, 32]. Previous studies have indicated that the multi-subunit nature of SIN3A provides unique contact surfaces for interaction with particular accessory proteins to regulate the transcription of specific genes [33]. For example, the association of the SIN3A-HDAC complex with Fam60a decreases histone acetylation at the cyclin D1 promoter and thus represses the expression of cyclin D1 [34]. The actions of SIN3A and STAT3 at the promoter of the tumor suppressor gene (TSG) can regulate cell survival by decreasing the expression of TSG [35]. The ZNF704/SIN3A complex represses a panel of genes, including *PER2*, resulting in the proliferation and invasion of breast cancer [36]. The SIN3A-HDAC complex is recruited to the promoter of *GATA3* by FOXN3 to repress *GATA3* expression, thus repressing breast cancer metastasis [37]. Moreover, LSD1 coordinates with the SIN3A/HDAC complex to maintain sensitivity to chemotherapy in breast cancer [38]. However, whether other proteins can bind to the SIN3A complex needs further investigation.

In this study, we found that the intracellular activated complement fragment C3b entered the nucleus to induce lung cancer resistance to PTX by repressing cell apoptosis. Mechanistically, nuclear C3b physically interacts with the SIN3A/HDAC complex to repress the expression of GADD45A by enhancing the association between SIN3A/HDAC and the GADD45A promoter.

## Results

### C3 is upregulated in PTX-resistant lung cancer cells

**Fig. 1:**
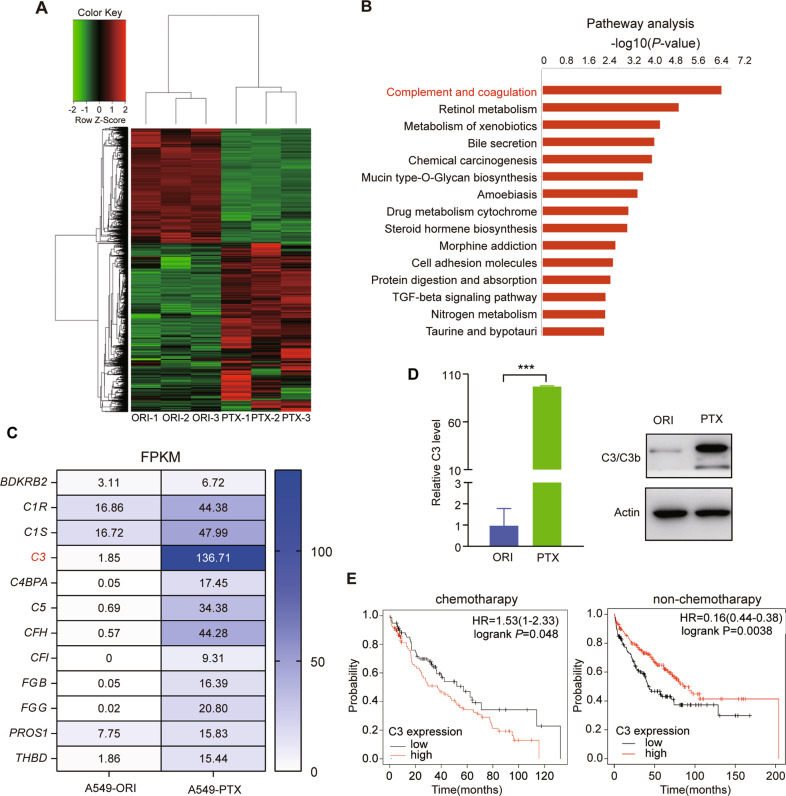
C3 is upregulated in PTX-resistant NSCLC A549 cells. **A** Heatmap of differentially expressed genes between A549-ORI and A549-PTX cells by RNA-Seq. **B** The top 15 enriched signaling pathways based on the 2914 upregulated genes in A549-PTX cells. **C** FPKM values of genes in the complement and coagulation cascade pathways. **D** C3 expression at the mRNA and protein levels in A549-ORI and A549-PTX cells measured by qRT‒PCR and immunoblotting (IB) assays. Data represent the mean ± SD, *n* = 3, two-tailed *Student’s t test*. **E** Kaplan‒Meier survival analysis for the relationship between the survival rate of chemotherapy-treated or nonchemotherapy-treated lung cancer patients and the expression of C3 (by log-rank). HR, hazard ratio. *P < 0.05, **P < 0.01, ***P < 0.001 and ****P < 0.0001.

Paclitaxel has been widely used for the treatment of NSCLC. It arrests exposed cells in the G2/M-phase by stabilizing the microtubule polymer, eventually leading to cell apoptosis. To study PTX resistance, we first treated original NSCLC A549 cells (A549-ORI) with increasing doses of PTX to establish PTX-resistant cells (A549-PTX) (Fig. S1A). RNA-sequencing analysis was then performed to identify the dysregulated genes in PTX-resistant cells. The results showed that 2914 genes were upregulated and 1,974 genes were downregulated in A549-PTX cells (Fig. 1A). Furthermore, the gene enrichment analysis for these dysregulated genes showed that the complement and coagulation pathway was the most upregulated pathway in PTX-resistant cells (Fig. 1B), in which the core complement component *C3* displayed the abundant and most upregulated expression level (Fig. 1C). The results of qRT‒PCR and immunoblotting assays for C3 further supported this finding at both the mRNA and protein levels (Fig. 1D). In addition, KM-plot survival analysis revealed that a higher C3 level indicated shorter survival in lung cancer patients who received chemotherapy, while a higher C3 level indicated longer survival in lung cancer patients who did not receive chemotherapy (https://kmplot.com) (Fig. 1E), suggesting the dual role of complement in different contexts of cancer [39]. Therefore, these results suggest that the C3 expression level is highly correlated with PTX resistance in lung cancer.

### C3 is required for PTX resistance in NSCLC

**Fig. 2:**
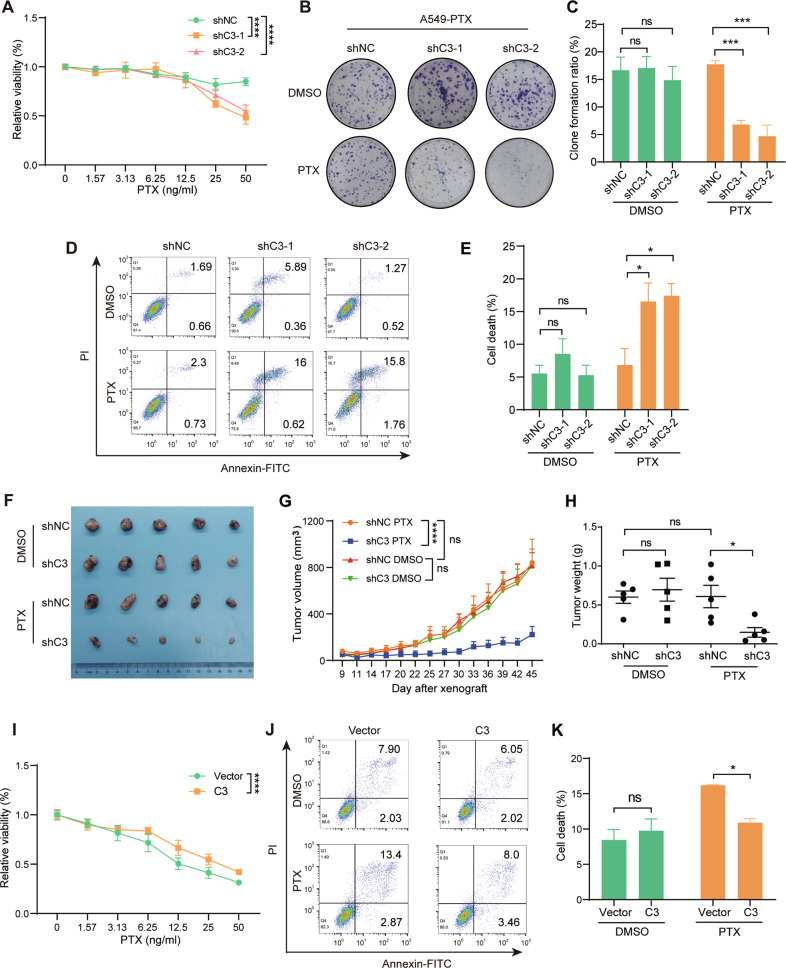
C3 is required for PTX resistance in A549 cells. **A** C3 insufficiency enhanced PTX cytotoxicity in A549-PTX cells, as demonstrated by the CCK8 assay. Data represent the mean ± SD, *n* = 5, two-tailed Student’s *t test*. C3 insufficiency reduced the colony formation ratio of A549-PTX cells with PTX treatment. Images (**B**) and quantitative results (**C**). Data represent the mean ± SD, *n* = 3, two-tailed *Student’s t test*. C3 insufficiency promoted PTX-induced apoptosis in A549-PTX cells, as detected by FCM analysis. Scatter plot (**D**) and quantitative results (**E**). FCM, flow cytometry. Data represent the mean ± SD, *n* = 3, two-tailed Student’s *t test*. **F**–**H** C3 insufficiency increased the sensitivity of A549-PTX cells to PTX treatment in tumor-bearing mice. Images (**D**), growth curve (**E**) and tumor weight (**F**). Data represent the mean ± SEM, *n* = 5, two-tailed Student’s *t test*. **I** Ectopic C3 expression impeded PTX cytotoxicity in A549-ORI cells, as demonstrated by the CCK8 assay. Data represent the mean ± SD, *n* = 5, two-tailed Student’s *t test*. **J**–**K** Ectopic C3 expression suppressed PTX-induced apoptosis in A549-ORI cells, as detected by FCM analysis. Data represent the mean± SD, *n* = 3, two-tailed Student’s *t test*. ns, no significance, *P < 0.05, **P < 0.01, ***P < 0.001 and ****P < 0.0001.

To identify the biological role of C3 in lung cancer resistance to PTX, we knocked down C3 expression in A549-PTX cells (Fig. S1B) and found that the viability and colony formation capability of the C3-insufficient cells were dramatically decreased after PTX treatment (Fig. 2A, B and C). Meanwhile, PTX-induced apoptosis significantly increased after knockdown of C3 (Fig. 2D and E). The in vivo data further demonstrated that C3 insufficiency dramatically suppressed tumor growth with PTX treatment but had no effect without PTX treatment (Fig. 2F, G, H). In contrast, ectopic expression of C3 in A549-ORI cells (Fig. S1C) significantly increased cell viability with PTX treatment (Fig. 2I), which was further supported by the significantly decreased apoptotic cells (Fig. 2J, K). Together, these results indicate that C3 is required for NSCLC resistance to PTX.

### The C3 cleavage product C3b translocates into the nucleus in PTX-resistant cells

It has been shown that C3 promotes ovarian cancer cell proliferation by activating the C3a-C3aR-PI3K-AKT signaling pathway [22], and the PI3K/AKT pathway is a key link to modulate drug resistance [40]. Thus, we tested whether C3a/C3aR may mediate PTX resistance via the PI3K/AKT pathway in NSCLC. However, we found that C3aR was abundantly present only inside cells but not on the cell membrane in both A549-ORI and A549-PTX cells (Fig. S2A). Moreover, we found that the expression level of C3aR in A549-PTX cells was much lower than that in A549-ORI cells (Fig. S2B). Therefore, C3 regulated PTX resistance in NSCLC unlikely via the C3a-C3aR-PI3K-AKT signaling pathway.

**Fig. 3:**
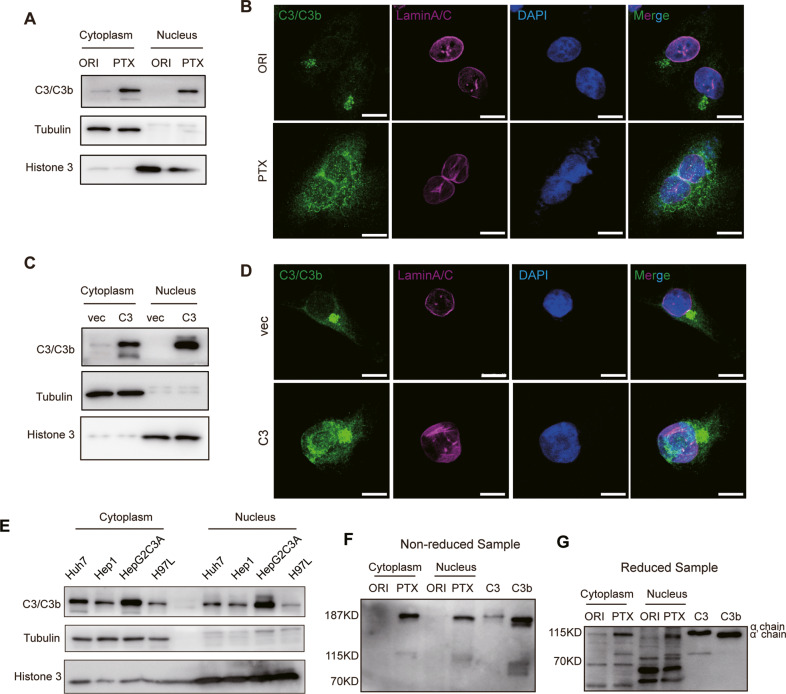
C3b may translocate into the nucleus. C3/C3b subcellular distribution detected by immunoblotting (**A**, **C**) and immunocytochemistry (**B**, **D**) assays in A549-ORI and A549-PTX (**A**, **B**) or A549 cells with or without ectopic C3 expression (**C**, **D**). Scale bar, 10 μm. **E** C3b is also located in the nucleus of hepatocellular cells. Cytoplasmic and nuclear distribution of C3/C3b in HepG2C3a, H97 L, Huh7, and Hep1 cells detected by immunoblotting assay with indicated antibodies. C3b may translocate into the nucleus of A549-PTX cells. C3 and C3b distribution in the cytoplasm and nucleus of A549-ORI and A549-PTX cells detected by immunoblotting assay in nonreduced (**F**) and reduced (**G**) conditions. Purified C3 (~190 kDa) and C3b (~181 kDa) were used as controls. During C3 activation, the α chain of C3 is cleaved C3a away, thus being named the α’ chain, and the remaining main fragment is C3b.

To further explore the molecular mechanism whereby C3 regulates PTX resistance in NSCLC, we first examined C3 expression and location in the cytoplasm and nucleus of A549-ORI and A549-PTX cells. C3 was detected only in the cytoplasm in A549-ORI cells but was highly expressed both in the cytoplasm and nucleus of A549-PTX cells (Fig. 3A, B). The ectopic expression of C3 in A549-ORI cells also replicated this finding (Fig. 3C, D). The nuclear location of C3 was also observed in hepatocytes, such as HepG2C3a, Hep1, H97L and Huh7 cell lines, which are the major source for the biosynthesis of complement components, including C3 (Fig. 3E), indicating the potential biological function of C3 in the nucleus. C3 is a macromolecule with a molecular weight of ~190 kDa, leading to difficulty in free nuclear transport. It is a typical approach for nucleus-transported proteins using nuclear localization signal (NLS)-dependent and cargo protein-mediated methods. We first predicted the NLS of C3 on an NLS-mappers website (https://nls-mapper.iab.keio.ac.jp/cgi-bin/NLS_Mapper_form.cgi) (Fig. S3A) and mutated this sequence to detect C3 nuclear translocation. The result showed that this mutation could not block C3 nuclear translocation (Fig. S3B), indicating an NLS-independent manner for C3 nuclear transport. To exert biological functions, extracellular intact C3 needs to be proteolytically cleaved into small fragment C3a (~8.5 kDa) and large fragment C3b by typical C3 convertases, such as C4bC2a and C3bBb, or by other proteases, such as plasmin and thrombin [10]. Next, using purified C3 and C3b as positive controls, we employed an immunoblotting assay to identify which form of C3 was transported into the nucleus. The results showed that the nuclear form was C3b but not C3, and intact C3 was retained in the cytoplasmic fraction of A549-PTX cells (Fig. 3F). Under reduced conditions, C3 and C3b may produce α (115 kDa) or α’ chains in addition to β chains, respectively, since C3a is cleaved from α chains to produce α’ chains [10]. Consistently, the α chain and α’ chain were observed in the cytoplasm and nucleus of A549-PTX cells after β-mercaptoethanol treatment, respectively (Fig. 3G). Therefore, the cleaved C3b fragment but not the intact C3 translocated into the nucleus in PTX-resistant NSCLC cells. In addition, to identify the protease responsible for intracellular C3 cleavage in A549-PTX cells, we knocked out the expression of cathepsin L (CTSL) (Fig. S3C), which cleaves C3 to generate C3a inside T cells [16]. However, CTSL deficiency failed to inhibit C3b entry into the nucleus (Fig. S3D), indicating that proteases other than CTSL cleaved C3 to produce C3b in A549-PTX cells.

### Nuclear C3b assembles with the SIN3A complex in PTX-resistant cells

**Fig. 4:**
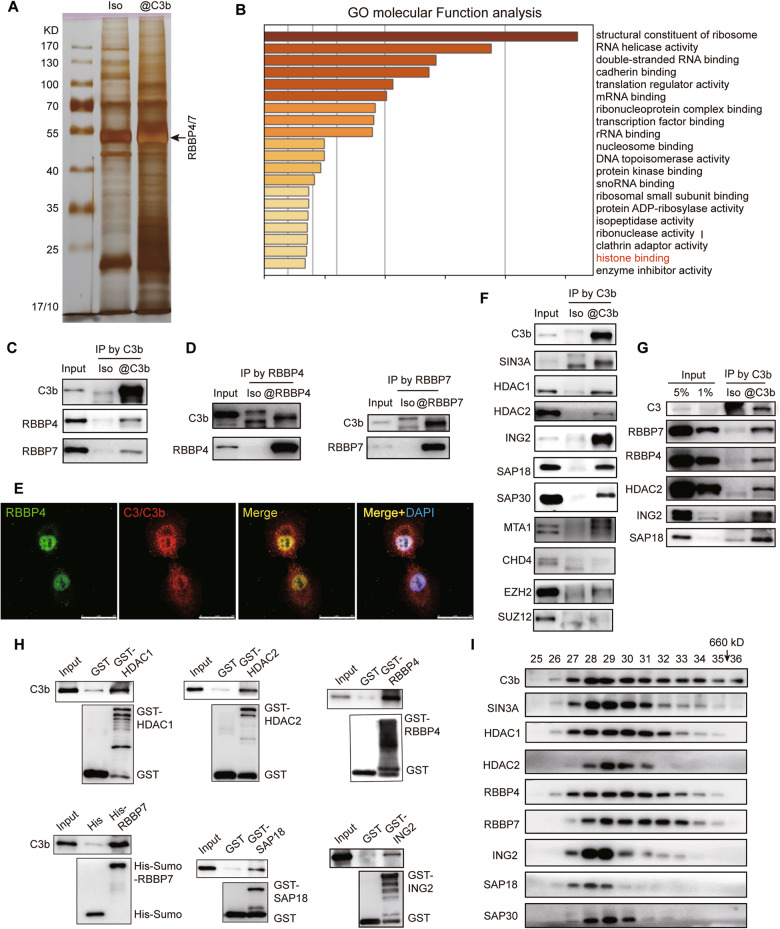
Nuclear C3b assembles with the SIN3A complex in PTX-resistant cells. **A** Silver staining of C3b-binding nuclear proteins in A549-PTX cells prepared by Co-IP. **B** The top 20 enriched signaling pathways based on the 195 C3-binding nuclear proteins in A549-PTX cells. Verification of the interaction between C3b and RBBP4/7 by mutual Co-IP assays with anti-C3/C3b antibody (**C**) or anti-RBBP4/7 antibodies (**D**) or by immunocytochemistry assay (**E**) in A549-PTX cells. Scale bar, 25 μm. **F** C3b interacted with RBBP4/7-containing SIN3A complex but not NuRD or PRC2 complex in A549-PTX cells determined by Co-IP assay using anti-C3/C3b antibody followed by immunoblotting assay using the indicated antibodies. The SIN3A complex contains SIN3A, HDAC1/2, RBBP4/7, SDS, ING2, SAP18 and SAP30 subunits, while the NuRD complex contains MTA1 and CHD4 subunits, and the PRC2 complex contains EZH2 and SUZ12 subunits. **G** The interaction of C3b with the SIN3A complex was further confirmed by Co-IP and immunoblotting assays in A549-ORI cells with ectopic C3 expression. **H** A GST pull-down assay validated the interaction of C3b with the subunits of the SIN3A complex. **I** Identification of the physiological interaction of C3b and the SIN3A complex in A549-PTX cells by gel chromatography and immunoblotting. C3b assembles a new complex with the SIN3A complex containing SIN3A, HDAC1/2, RBBP4/7, ING2 and SAP18/30. The fraction numbers and elution of molecular weight markers with arrows are indicated.

Considering that both A549-ORI and A549-PTX cells expressed C3 in the cytoplasm but only A549-PTX cells expressed C3b in the nucleus, we next harvested nuclear proteins to identify the C3b-binding proteins in A549-PTX cells with a co-IP assay followed by mass spectrometric analysis (Fig. 4A). Based on the identified proteins, the GO analysis revealed that C3b-interacting proteins were mainly associated with the regulation of gene expression (Fig. 4B). C3/C3b have been reported to bind to histones by ELISA [21]; thus, we chose the histone-binding protein RBBP4/7 for further exploration (Fig. 4B). Using a mutual co-IP assay, we determined the interaction between C3b and RBBP4/7 (Fig. 4C and D), which was further verified by the colocalization of C3b and RBBP4/7 in the nucleus of A549-PTX cells with an IF assay (Fig. 4E). Thus, these results indicate that C3b interacts with RBBP4/7 in the nucleus of PTX-resistant NSCLC cells.

It has been reported that as core components, RBBP4/7 are involved in the assembly of distinct complexes, such as NuRD, SIN3A and PRC2 [41]. To further identify which RBBP4/7-containing complex could interact with C3b in A549-PTX cells, we performed a co-IP assay with antibodies against C3b to capture SIN3A, HDAC1/2, RBBP4/7, SDS, ING2, SAP18 and SAP30 (subunits of SIN3A complex); MTA1, CHD4 (subunits of NuRD complex); EZH2, or SUZ12 (subunits of PRC2 complex). The results showed that C3b only interacted with the subunits of the SIN3A complex but not the subunits of either the NuRD or PRC2 complexes (Fig. 4F). The interaction between C3b and the SIN3A complex was also validated by the ectopically expressed C3 in A549-ORI cells with a co-IP assay (Fig. 4G). The pull-down assay with purified protein C3b and bacterially expressed GST-HDAC1/2, -RBBP4, -SAP18, -ING2 and His-Sumo-RBBP7 further revealed that C3b could interact with RBBP4/7, HDAC1/2, SAP18 and ING2 directly (Fig. 4H). To further detect the physiological interaction of C3b with the SIN3A complex, nuclear proteins extracted from A549-PTX cells were fractionated by size exclusion using gel chromatography. We found that native C3b eluted with a much greater apparent molecular mass than monomeric C3b. In an immunoblotting assay, C3b was detected in chromatographic fractions greater than 660 kDa (fraction 35), which appeared simultaneously with the subunits of the SIN3A complex mainly in fractions 28 and 29 (Fig. 4I), indicating that C3b interacts with the SIN3A complex to form a novel complex with a very large molecular weight. Together, these results demonstrate that C3b assembles a complex with SIN3A in the nucleus of PTX-resistant NSCLC cells.

### Identification of genome-wide transcriptional targets for the C3b-containing SIN3A complex

**Fig. 5:**
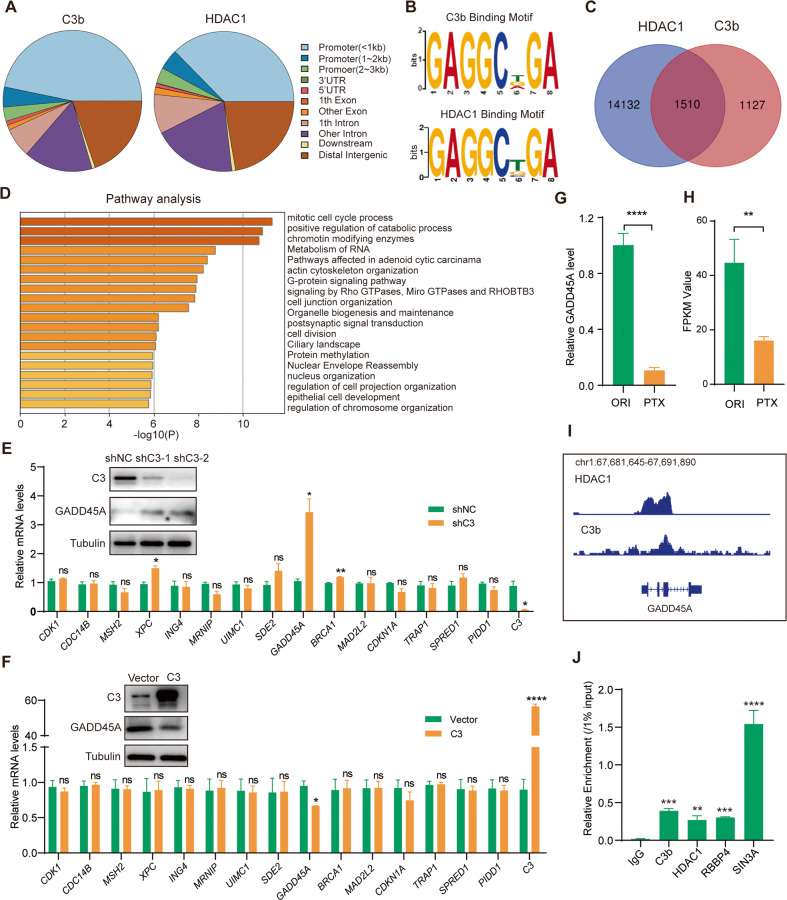
Identification of *GADD45A* as a C3b/SIN3A complex target gene by genome-wide transcriptional analysis. **A** Genomic distribution of the transcriptional targets of C3b and HDAC1 based on ChIP-Seq data. **B** DNA binding motifs of C3b and HDAC1 by MEME-ChIP analysis. **C** Venn diagrams of overlapping genes targeted by C3b and HDAC1 in A549-PTX cells. **D** Pathway analysis of 1,510 overlapping genes targeted by C3b and HDAC1 in (**C**). The mRNA levels of the indicated genes as measured by qRT‒PCR in C3-insufficient A549-PTX cells (**E**) and C3-overexpressing A549-ORI cells (**F**). The protein levels of C3 and GADD45A are shown in the related figures. The GADD45A mRNA level in A549-ORI and A549-PTX cells measured by qRT‒PCR (**G**) or RNA-Seq (**H**). **I** The binding profiles of C3b and SIN3A on the *GADD45A* promoter. **J** Verification of the binding of the C3b/SIN3A complex to the *GADD45A* promoter by ChIP/qRT‒PCR assay using antibodies against the indicated proteins in A549-PTX cells. Data represent the mean ± SD, *n* = 3, two-tailed Student’s t test. *P < 0.05, **P < 0.01, ***P < 0.001 and ****P < 0.0001.

To explore the functional significance of the interaction of C3b with the SIN3A complex, we next analyzed the genome-wide transcriptional targets of this C3b-containing SIN3A complex. Chromatin immunoprecipitation (ChIP)-based deep sequencing (ChIP-seq) was performed in A549-PTX cells using antibodies against C3b and HDAC1, the core factor of the SIN3A complex. A quantitative Venn diagram analysis confirmed the similar distribution of C3b and HDAC1 target sites, which were mainly located in the promoter region (Fig. 5A). Analysis of the characteristic genomic signatures of C3b and HDAC1 showed 15 and 26 discriminative regular expression motif elicitation (DREME), respectively, among which three DREME exhibited a very similar binding motif (Fig. 5B, Fig. S6A and B). These results strongly support that nuclear C3b exerts its transcriptional regulatory function at least by assembling a novel complex with the HDAC1-containing SIN3A complex.

The data from anti-C3b ChIP-Seq and anti-HDAC1 ChIP-Seq were then cross-analyzed to identify overlapping DNA sequences, which were considered to be the targets of the C3b-containing SIN3A complex (Fig. 5C). These 1,510 overlapping target genes were classified into different biological processes by expression analysis with the Metascape website (https://metascape.org/gp/index.html), among which the mitotic cell cycle process pathway was enriched most obviously (Fig. 5D). Next, the expression levels of the genes in the mitotic cell cycle pathway were verified by qRT‒PCR and/or immunoblotting assays. The results showed that C3 insufficiency in A549-PTX cells resulted in a significant increase in *XPC*, *GADD45A* and *BRCA1* (Fig. 5E), whereas ectopic expression of C3 in A549-ORI cells only inhibited *GADD45A* expression (Fig. 5F). As expected, *GADD45A* was downregulated in A549-PTX cells compared with A549-ORI cells, as determined by qRT‒PCR (Fig. 5G) and RNA-Seq (Fig. 5H). Moreover, C3 and HDAC1 exhibited similar peak locations on the *GADD45A* promoter (Fig. 5I). Moreover, a ChIP assay combined with qPCR using specific primers in the *GADD45A* promoter region revealed strong enrichment of C3, HDAC1, RBBP4 and SIN3A on the promoter of *GADD45A* (Fig. 5J). Therefore, these results identified *GADD45A* as one of the downstream target genes of the C3b-SIN3A complex in PTX-resistant NSCLC cells.

### C3b downregulates *GADD45A* expression by enhancing the binding of the SIN3A complex to the *GADD45A* promoter

**Fig. 6:**
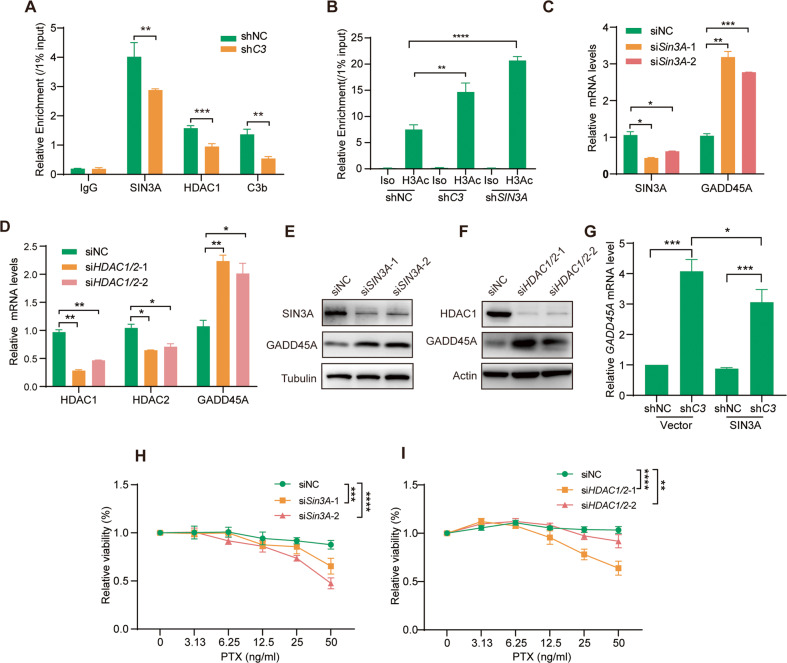
C3b downregulated *GADD45A* transcription by enhancing the binding of the SIN3A complex with the *GADD45A* promoter. **A** C3 insufficiency reduced the binding of the C3b/SIN3A complex to the GADD45A promoter in A549-PTX cells, as detected by ChIP/qRT‒PCR assays using antibodies against SIN3A, HDAC1 or C3/C3b. **B** Insufficiency of C3 or SIN3A increased the acetylation of histone 3 in the *GADD45A* promoter region, as determined by ChIP/qRT‒PCR assays using an antibody against panacetylated histone 3 (H3Ac). Downregulation of the SIN3A complex promoted GADD45A expression. Insufficiency of either SIN3A (**C,** **E**) or HDAC1 (**D**, **F**) enhanced GADD45A expression at the mRNA (**C**, **D**) and protein (**E**, **F**) levels in A549-PTX cells, which were measured by qRT‒PCR and immunoblotting, respectively. G. Ectopic expression of SIN3A impaired the effect of C3 insufficiency on enhancing *GADD45A* transcription in A549-PTX cells, as determined by qRT-PCR. **H** Insufficiency of either SIN3A enhanced PTX-induced cytotoxicity in A549-PTX cells, as determined by CCK8 assay. **I** Insufficiency of either HDAC1/2 enhanced PTX-induced cytotoxicity in A549-PTX cells, as determined by CCK8 assay. Data represent the mean ± SD, n = 3 (**A**–**D**, **G**) or 5 (**H**), two-tailed Student’s *t* test. *P < 0.05, **P < 0.01, ***P < 0.001 and ****P < 0.0001.

To further explore the mechanism by which C3 regulates the expression of *GADD45A* via the SIN3A complex, we knocked down C3 expression and detected the binding of the C3b-SIN3A complex to the *GADD45A* promoter region using the ChIP and qRT‒PCR assays mentioned above. The results showed that C3 insufficiency significantly reduced the recruitment of SIN3A, HDAC1 and C3b to the *GADD45A* promoter (Fig. 6A). Moreover, C3 insufficiency did not change the expression of SIN3A subunits, including SIN3A, RBBP4/7, HDAC1/2 and SAP18, at the mRNA and protein levels (Fig. S4A and B). This result suggests that additional C3 in the SIN3A complex may enhance the binding activity of the SIN3A complex to the *GADD45A* promoter region. Using a similar approach, we observed that the level of pan-H3 acetylation (H3Ac) was markedly increased at the *GADD45A* promoter region upon knockdown of *C3* or *SIN3A* (Fig. 6B), indicating that as a transcription suppressor, the C3b-SIN3A complex suppresses *GADD45* expression by inhibiting histone acetylation of the *GADD45D* promoter region. Significantly, knockdown of either SIN3A or HDAC1 resulted in upregulation of GADD45A at both the mRNA (Fig. 6C, D) and protein levels (Fig. 6E, F). The upregulated expression of GADD45A upon C3 knockdown was constrained when SIN3A was overexpressed in A549-PTX-shC3 cells (Fig. 6G), indicating that C3 repressed GADD45A expression via the SIN3A complex. Importantly, knockdown of SIN3A or HDAC1 sensitized A549-PTX cells to PTX treatment (Fig. 6H and I). Together, these findings revealed that C3 repressed GADD45A expression by enhancing the binding of SIN3A complex with the *GADD45A* promoter region, thus involved in the development of PTX resistance.

### GADD45A is involved in PTX resistance in NSCLC cells

**Fig. 7:**
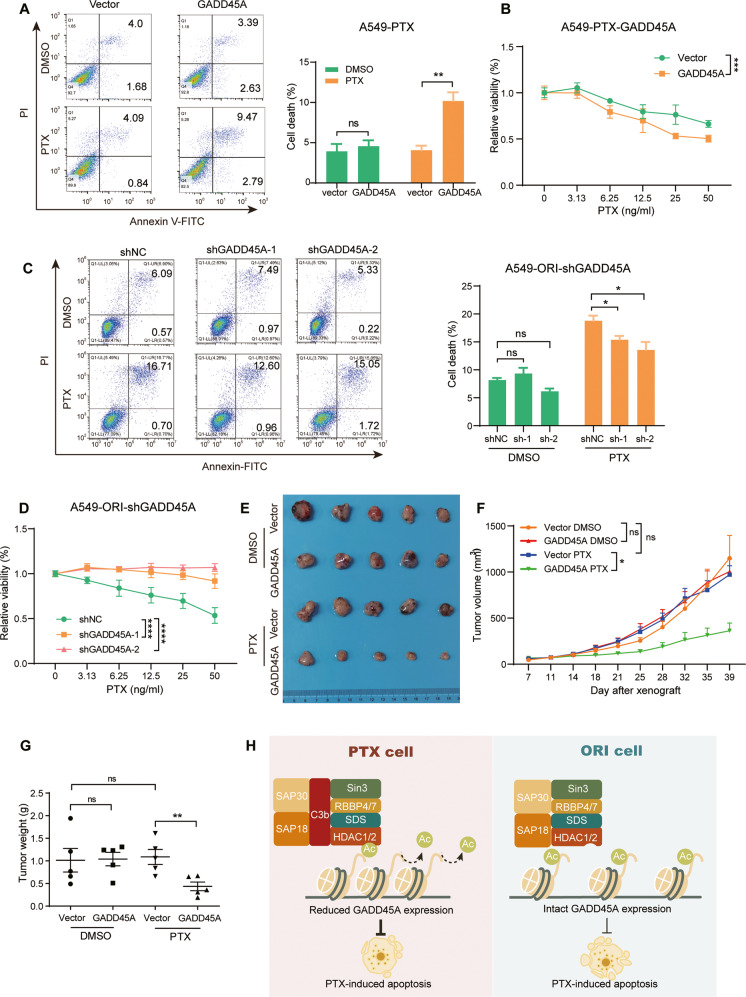
C3-SIN3A-GADD45A is involved in the apoptosis of A549-PTX cells. The effect of GADD45A expression level on apoptosis and cell proliferation. Ectopic GADD45A expression enhanced, while GADD45A insufficiency suppressed apoptosis induced by PTX treatment in A549-PTX (**A**) and A549-ORI (**C**) cells. Consistently, the related cell proliferation was inhibited (**B**) or enhanced (**D**) in the presence of PTX. Apoptotic cells were stained by FCM analysis with Annexin V-FITC/PI, and cell viability was measured by CCK8 assay. Data represent the mean ± SD, *n* = 3 (**A**, **C**) or 5 (**B**, **D**), two-tailed Student’s *t* test. Ectopic GADD45A expression enhanced the sensitivity of A549-PTX cells to PTX treatment in tumor-bearing mice. Tumor images (**E**), tumor growth curve (**F**) and tumor weight (**G**). Data represent the mean ± SEM, *n* = 5, two-tailed Student’s *t* test. **H** The working model of nuclear C3b in promoting resistance to PTX by interacting with the SIN3A complex on the promoter region of targeted genes, including *GADD45A*.

GADD45A is involved in cell cycle arrest, apoptosis and DNA repair in response to a variety of stresses [42]. To assess its function in the regulation of NSCLC PTX resistance, we established stable GADD45A-overexpressing A549-PTX cells, which was confirmed by qRT‒PCR (Fig. S5A). Then, we treated these cells with PTX to detect PTX-induced apoptosis. The results showed that ectopic GADD45A expression decreased the resistance to PTX by promoting apoptosis (Fig. 7A), which was further supported by the impeded cell viability by ectopic GADD45A expression (Fig. 7B). In contrast, the GADD45A-insufficient A549-ORI cells (Fig. S5B) were resistant to PTX-induced apoptosis compared to the GADD45A-sufficient cells (Fig. 7C), thus obtaining a growth advantage over the GADD45A-sufficient cells under PTX treatment (Fig. 7D). Moreover, using the xenograft mouse model, we corroborated that ectopic GADD45A expression could significantly sensitize A549-PTX cells to PTX treatment (Fig. 7E–G). Taken together, these results reveal that impaired GADD45A expression by the C3b-SIN3A complex is involved in the development of PTX resistance in NSCLC cells.

## Discussion

In the current study, we reveal that nuclear C3b acts as a cofactor of the chromatin remodeling SIN3A complex to repress the expression of GADD45A, thus conferring acquired resistance of lung cancer to PTX treatment. This study is a first step toward elucidating that tumor cell-derived complement proteins enter the nucleus to induce chemoresistance via epigenetic regulation. This finding also suggests that C3 may act as a predictor for drug resistance in NSCLC patients and might be a potential target for NSCLC chemotherapy.

Previous studies have demonstrated that C3 regulates tumor initiation and progression through the C3a-C3aR-PI3K-AKT signaling pathway. The binding of tumor cell-secreted C3a to membrane C3aR activates the PI3K-AKT pathway, thus facilitating ovarian cancer cell proliferation [22]. C3a-C3aR-PI3K-AKT signaling also induces cancer-associated fibroblasts to secrete pro-metastatic cytokines and to express extracellular matrix components in breast cancer [23] and modulates tumor-associated macrophages to repress the antitumor immunity of PD-L1 antibody [43]. However, we detected little expression of C3aR on the plasma membrane either in original or PTX-resistant NSCLC cells. PTX-resistant cells even expressed lower C3aR levels than the original NSCLC cells. Moreover, C3 knockdown reversed the sensitivity of tumor cells to PTX treatment and had no effect on proliferation, indicating that C3 regulated NSCLC PTX resistance in a C3a/C3aR/PI3K/AKT-independent manner.

Complement has been known to be activated almost exclusively in the blood, and its components are synthesized by the liver and restricted cell types. This classical paradigm has changed until the intracellular complement components in immune cells were discovered in recent years [16, 44]. It is well established that many types of cells, including immune cells, epithelial cells, fibroblast cells and endocrine cells, are capable of synthesizing and activating complement proteins [16, 17, 23]. This locally operating intracellular “complosome” is an important player in driving vital cellular processes, such as immune response, metabolism, and proliferation [6, 17, 18, 45]. In addition, serum-derived C3/C3a, but not C3b, may enter the nucleus to bind to histone proteins in B cells, suggesting a potential ability to induce chromatin remodeling and regulate DNA transcription [21]. Herein, we revealed that C3 was upregulated in PTX-resistant NSCLC cells, and its active fragment C3b translocated into the nucleus to regulate gene transcription indirectly by interacting with the SIN3A/HDAC corepressor complex; thus, the subsequent downregulation of GADD45D induced PTX resistance. Moreover, the nuclear translocation of C3b was also found in hepatocellular carcinoma cells, a dominant tissue/cell type responsible for C3 production. This finding suggests that nuclear C3b, another subcellular distribution of the C3 cleavage product, exerts a complement-independent function, at least in epigenetically regulating gene expression.

In both yeast and mammals, together with diverse interacting partners, SIN3 proteins have been shown to activate or repress the transcription of target genes for regulating various biological functions [33], in which the repression activity mostly involves deacetylation of histone or non-histone proteins, while the activation process is less clear and may be HDAC independent [33, 46]. For example, the interaction between SMAR1 and the SIN3A/HDAC complex contributes to cyclin D1 repression via a histone deacetylation-related mechanism [47]. The recruitment of wild-type p53 to the SIN3A/HDAC complex represses the expression of target genes such as *Map4* [48], while Ebp1 interacting with SIN3A/HDAC shows a strong repressive effect on E2F1 transcription [49]. The lncRNA NEAT1 scaffolds the binding of FOXN3 to the SIN3A/HDAC complex, thus repressing *GATA3* transcription [37]. In yeast, Sin3 (also called GAM2) may instead act as a positive transcriptional regulator of GAM3/ADR6 [50]. Yeast histone deacetylase Rpb3 may be recruited by MAPK Hog1 upon osmotic stress to assemble the Rpd3-Sin3 complex onto the promoters of osmo-responsive genes, thus inducing their transcription [51]. Interestingly, recruitment of the mSin3A/HDAC complex by p53 in differentiated cells downregulates Nanog, while Sox2 association with the mSin3A/HDAC complex activates Nanog in proliferating embryonic stem cells [52, 53]. In the present study, we demonstrate that nuclear C3b may scaffold the SIN3A/HDAC complex to repress the expression of GADD45A, thus inducing NSCLC resistance to PTX.

The growth arrest and DNA damage (*GADD*)-inducible gene family is often upregulated in response to various environmental stresses and drug therapies. *GADD45*A was the first stress-inducible gene found to be activated by the p53 tumor suppressor [54]. Moreover, GADD45A was demonstrated to be involved in many cellular and biological processes, such as DNA damage, cell proliferation, cell cycle and apoptosis [54–56]. Our study provides a p53-independent mechanism for GADD45A regulation, in which nuclear C3b interacts with the SIN3A/HDAC complex to repress GADD45A transcription via histone deacetylation in the promoter region of GADD45A in PTX-resistant NSCLC cells. Similarly, methylation-mediated silencing of GADD45A also contributes to prostate cancer resistance to docetaxel [57], indicating that both deacetylation and methylation of the *GADD45A* promoter region are involved in the transcriptional regulation of *GADD45A*. Moreover, it has been reported that the histone deacetylase inhibitor trichostatin A (TSA) could increase GADD45A expression to stimulate the cell cycle checkpoint and induce apoptosis, which provides a potential strategy for cancer treatment [58]. Herein, our study further elucidates the underlying mechanism by which the C3b/SIN3A/HDAC complex downregulates the expression level of GADD45A via histone deacetylation.

Complement C3 mainly circulates in the blood in an inactive form and is also expressed intracellularly in various cell types, including immune cells [16] and epithelial cells [59]. To exert its function, C3 needs to be cleaved into biologically active C3a and/or C3b in a convertase-dependent (for extracellular C3) or -independent (for intracellular C3) way. Intracellular C3 in T cells is cleaved by CTSL in lysosomes and endosomes to C3a and C3b, and C3a is required for homeostatic T cell survival by binding to C3aR [16]. However, we found that CTSL deficiency had no effect on the nuclear C3b level in PTX-resistant A549 cells, indicating that CTSL is another protease responsible for C3 cleavage. The other key step involves how C3b is translocated into the nucleus. Due to the large size of C3b (~180 kDa), this process most likely requires the assistance of nuclear transporters. However, mutation of the predicted nuclear localization signal (NLS) sequence in C3b failed to block its nuclear translocation. Therefore, the underlying mechanisms for C3b nuclear transportation require further investigation.

In summary, our study revealed that the tumor cell-derived activated fragment C3b can be translocated into the nucleus to epigenetically regulate gene transcription by interacting with the chromatin remodeling SIN3A/HDAC complex. This new C3b/SIN3A/HDAC corepressor complex may suppress the transcription of downstream target genes, such as *GADD45A*, by deacetylating histone 3 around its promoter region, thus inhibiting apoptosis induced by PTX and leading to PTX resistance in NSCLC cells. Therefore, we established a link between the nuclear complement system and epigenetic regulation of genes and further revealed its function in cancer chemoresistance.

## Materials and methods

### Cell culture and treatment

HEK-293T, HepG2C3a, Hep1, Huh7, H97 L, A549 cells were obtained from American Type Culture Collection (ATCC). HEK-293T, HepG2C3a, Hep1, Huh7 and H97 L were cultured in DMEM and the A549 cells were cultured in RPMI-1640 medium. All cell media were supplemented with 10% fetal bovine serum and antibiotics at 37 °C in an atmosphere of 5% CO_2_.

To establish A549 cells resistant to paclitaxel (PTX), A549 cells were exposed to an initial PTX concentration of 5 ng/ml in RPMI-1640 supplemented with 10% fetal bovine serum and antibiotics for 2 weeks. The concentration of PTX then sequentially increased to 10 ng/ml (2 week), 20 ng/ml (2 week), 40 ng/ml (2 week), 80 ng/ml (2 week), 160 ng/ml (2 week) and 320 ng/ml, in which the survived subpopulation in the highest PTX concentration was termed A549-PTX cells. The original A549 cells (A549-ORI) were passaged in the same manner without PTX treatment.

### Animal model

Animal care was performed with approval of the Institutional Animal Care and Use Committee at Shanghai Medical College, Fudan University. Six-week-old male nude mice were housed in laminar flow cabinets under specific pathogen-free conditions with food and water provided ad libitum. A total of 5 × 10^6^ A549-PTX cells were subcutaneously seeded in the left or right flank of the axilla of each mouse (each group contained 5 mice). When the tumors reached to 150 mm^3^ in volume, PTX (10 mg/kg) dissolved in buffer (5% DMSO + 40% PEG 400 + 5% Tween 80 + ddH_2_O) or blank buffer was intraperitoneally injected twice per week. When the tumors of control group reached to ~1000 mm^3^ in volume, these mice were sacrificed, and all subcutaneous tumors were collected and weighed.

### CCK8 and colony formation assays

For the relative cell viability assay, cells were seeded into 96-well plates at a density of 5000 cells/well and treated with DMSO or escalating concentrations of PTX from 1.57 ng/ml to 50 ng/ml. After 48 h, 10 µL of Cell Counting Kit-8 solution (CCK8, Yeasen Biotechnology, Shanghai, China) was added to each well and the samples were incubated at 37 °C for 120 min, and then absorbance was detected at 450 nm. The absorbance values of DMSO groups were considered as control, the relative value of PTX-treating groups (including 1.57, 3.13, 6.25, 12.5, 25, 50 ng/ml) to control were considered as the relative cell viability (%). The cell proliferation curves were constructed according to the OD450 value.

For the colony formation assay, cells were seeded into six-well plates at a density of 500 cells/well and treated with DMSO or PTX (100 ng/ml) on the twelfth day. After two days, the medium was discarded, and the cells were fixed with 4% paraformaldehyde for 30 min at room temperature and stained with 0.5% crystal violet. After washing with ddH_2_O for several times. Colonies were counted, and the colony formation ratio was calculated as follows: colony formation ratio = (colonies formed/cells seeded) × 100%.

### Apoptosis assay

Apoptotic stress after PTX treatment was measured by Annexin V/propidium iodide staining using an Annexin V-FITC/PI apoptosis kit (MultiSciences Biotech, Hangzhou, China). Annexin V-FITC was detected as green fluorescence, and propidium iodide staining was detected as red fluorescence.

### GST-pull down

GST-tagged HDAC1, HDAC2, SAP18 and ING2 were induced to be expressed in Rosetta (DE3) cells (Yeasen, Shanghai, China), and competent cells were lysed by an ultrasonic instrument. Then, the supernatant or the purified GST-RBBP4 (cat#Ag6110, PROTEINTECH, Chicago, USA)/His-Sumo-RBBP7 (cat#CSB-EP621959HU, CUSABIO, Wuhan, China) was incubated with GSTSep Glutathione Agarose Resin (Yeasen, Shanghai, China) for 3 h at 4 °C. Subsequently, purified complement component 3b (Complement Technology, USA) was incubated with the above mixture for 4 h at 4 °C. Wash the above Glutathione Agarose Resin After with PBST (0.02% Tween-20 in PBS), the protein complex was denatured with reducing SDS-loading buffer and heated at 100 °C for 10 min. The IB assay was then performed to detect the relevant proteins.

### Statistical analysis

The data are presented as the mean ± SD or mean ± SEM of at least three experiments. General paired or unpaired t-tests tests were conducted using GraphPad Prism 8 (GraphPad Software, CA, USA). Statistical tests were justified as appropriate. *p*-values less than 0.05 were considered statistically significant (*p < 0.05, **p < 0.01, ***p < 0.001, ****p < 0.0001). For the animal model data, we randomly assigned animals to treatment groups and untreated group, and all analyses were completed by investigators who were blinded to the experimental groups.

## Supplementary information

Supplementary materials

Reproducibility checklist

Original Western Blot

Dataset 1

Dataset 2

Dataset 3

Dataset 4

## Data Availability

The authors declare that all relevant data of this study are available within the article or from the corresponding author on reasonable request.
