# Supplementary material for: Nuclear complement C3b promotes paclitaxel resistance by assembling the SIN3A/HDAC1/2 complex in non-small cell lung cancer
Source: Cell Death Dis. 2023 Jun 8;14(6):351. doi: 10.1038/s41419-023-05869-y (PMC10250389; doi:10.1038/s41419-023-05869-y)
Supplement: Supplementary file 1 — Supplementary materials [file 41419_2023_5869_MOESM1_ESM.docx]

**Nuclear complement C3b promotes paclitaxel resistance by assembling the SIN3A/HDAC1/2 complex in non-small cell lung cancer**

Xiaochao Wang^1#^, Yan Hao^1#^, Jianfeng Chen^2^, Peipei Ding^1^, Xinyue Lv ^1^, Danlei Zhou^1^, Ling Li^1^, Luying Li^1^, Yanqing Xu^1^, Yumeng Zhu^1^, Wei Zhang^1^, Lu Chen^1^, Tian Liao^3^, Xianghuo He^1, 4^, Qing-Hai Ji^3^, Weiguo Hu^1, 4*^

^1^ Fudan University Shanghai Cancer Center and Institutes of Biomedical Sciences, Shanghai Medical College, Fudan University, Shanghai 200032, China

^2^ State Key Laboratory of Oncology in South China, Collaborative Innovation Center for Cancer Medicine, Sun Yat-sen University Cancer Center, 651 East Dongfeng Road, Guangzhou, Guangdong, 510060, P. R. China

^3^ Department of Head and Neck Surgery, Fudan University Shanghai Cancer Center, Department of Oncology, Shanghai Medical College, Fudan University, Shanghai 200032, China

^4^ Key Laboratory of Breast Cancer in Shanghai, Fudan University Shanghai Cancer Center, Fudan University, Shanghai 200032, China

^#^ These authors contributed equally to this work

Running title：C3b-SIN3A complex regulates PTX resistance in NSCLC

*Correspondence to:

Weiguo Hu, Cancer Institute, Fudan University Shanghai Cancer Center, 270 Dong’an Road, Shanghai 200032, China.

Email: [weiguohu@fudan.edu.cn](mailto:weiguohu@fudan.edu.cn)

**Supplementary materials and methods**

*RNA interference*

Transient transfection was performed by using Lipofectamine 3000 (Invitrogen, Carlsbad, USA) according to the manufacturer’s instructions. The siRNAs specifically targeting SIN3A and HDAC1 and the siRNA control were purchased from GenePharma. All primer sequences are listed in Table S1.

*Plasmid constructs and stable cell line generation*.

Full-length human C3 and SIN3A cDNA were amplified from A549-PTX cells and were inserted into the pCDH-CMV-MCS-EF1-Puro cDNA cloning and expression lentivector (cat# CD510B-1, System Biosciences, Palo Alto, USA) to construct pCDH-C3 and pCDH-SIN3A. Full-length human HDAC1, HDAC2, SAP18, and ING2 cDNA were amplified from A549-ORI cells and inserted into pGEX-6p-1 (GE Healthcare, Marlborough, USA) to construct pGEX-6p-1-HDAC1, pGEX-6p-1-HDAC2, pGEX-6p-1-SAP18, and pGEX-6p-1-ING2 plasmids. All primer sequences are listed in Table S2.

The NLS (ATTKRRHQQT) of C3 was predicted by the NLS mapper website (https://nls-mapper.iab.keio.ac.jp/cgi-bin/NLS_Mapper_form.cgi). We mutated the amino sequence “KRRH” to “DEDE” and constructed the PCDNA 3.1-C3-Flag-NLSm plasmid by overlap PCR. The primer sequences are shown in Table S2.

The pLKO.1-TRC cloning vector (plasmid #10878, Addgene, Cambridge, USA) was employed to construct scramble (SCR), C3, GADD45A and SIN3A shRNA plasmids. 293FT cells were co-transfected with the pLKO.1 plasmid and pMD.2G and psPAX2 plasmids to generate C3 GADD45A and SIN3A knockdown lentiviruses, respectively. The lentivirus was subsequently added to A549-ORI or A549-PTX cell culture medium for 48 h. The cells transduced with lentivirus were selected by puromycin at concentrations from 2 μg/ml to 10 μg/ml. Information regarding the primers and shRNA oligonucleotide sequences are shown in Table S3.

The lenti-gRNA-puro (plasmid #52963, Addgene, Cambridge, USA) was employed to construct lenti-gCTSL-1, lenti-CTSL-2, lenti-gCTSL-3 and lenti-gRNA-NC. 293FT cells were co-transfected with the lenti-gRNA plasmids or lenti-Cas9-blast (plasmid #52962, Addgene, Cambridge, MA) plasmid and pMD.2G and psPAX2 plasmids to generate gRNA or Cas9 lentiviruses. The cas9 lentiviruses was firstly added to A549-PTX cells culture medium for 48 h and the cells were selected by blast (5 μg/ml). The cas9 stable cells were subsequently treated with gRNA lentiviruses for 48 h and were selected by puromycin at concentrations from 2 μg/ml to 10 μg/ml. Information regarding the gRNA oligonucleotide sequences are shown in Table S3.

*Cytoplasmic/nuclear protein extraction.*

Cytoplasmic extracts were prepared by suspending the cell pellets in Buffer I, which contained 20 mM Tris-HCl pH 7.5, 10 mM NaCl, 2 mM MgCl_2_ and 1% NP-40 for 15 min at 4 °C, and then centrifuging for 1 min at 5000 rcf. The supernatant was transferred to Eppendorf tubes. Next, an equal volume of protein extraction buffer supplemented with protease inhibitors and phosphatase inhibitors (Beyotime Biotechnology, Shanghai, China) was added, and the samples were incubated with rotation at 4 °C for 1 hour. The lysates were centrifuged for 10 min at 4 °C at 10000 rcf. The supernatants which contain nuclear protein were used for IP or IP-MS. However, nuclear-cytoplasm *extraction* for IB was performed according to the Nuclear Extract Kit (ACTIVE MOTIF, California, USA) user manual.

*Immunoblotting assay*

Cell proteins were harvested in protein extraction buffer supplemented with protease inhibitors and phosphatase inhibitors (Beyotime Biotechnology, Shanghai, China) and analyzed with a standard protocol for IB. The proteins of interest in IB were visualized using X-ray film or CCD camera imaging devices ImageQuant LAS 4000 (GE Healthcare, Marlborough, USA). The antibodies used are listed in Table S4.

*Quantitative real‐time PCR*

Total RNA was isolated and reverse-transcribed into cDNA using Nuclezol reagent (Macherey-Nagel, Düren, Germany) and PrimeScript™ RT Master Mix (TaKaRa, Tokyo, Japan). Relative RNA expression levels were measured by an ABI system (Thermo Fisher Scientific, Waltham, USA). The sequences for the gene-specific primers used are listed in Supplementary Table S 5. β-actin was employed as an internal control.

*RNA-seq*

Total RNA was extracted from A549-ORI and A549-PTX cells with TRIzol reagent (Invitrogen, Grand Island, NY). The total RNA from each group from 3 different passages was pooled separately. RNA sequencing (RNA-seq) and bioinformatics analysis were conducted by Shanghai Novelbio Ltd. according to their established procedures. We applied the DEseq algorithm to filter the differentially expressed genes after significance and false discovery rate (FDR) analyses under the following 14 criteria: (1) fold change >1 or fold change<–1 and (2) FDR <0.05 (Supplementary Data 1). Pathway analysis was used to identify significant pathways of the differentially expressed genes according to the KEGG database.

*Co-IP*

Cell proteins were prepared as described above and incubated with protein A/G-linked magnetic beads (Thermo Fisher Scientific, Waltham, USA) and specific antibodies overnight. Next, the complex was washed with protein extraction buffer and boiled with SDS-loading buffer containing β-mercaptoethanol (Amresco, Solon, USA). The co-IP samples were then subjected to LC/MS, IB analysis or silver staining. The antibodies used in the co-IP assays are listed in Table S4.

*Mass spectrometry*

The IP sample were prepared as described above and the mass spectrometry analysis was conducted by Shanghai Institute of Materia Medica Chinese Academy of Sciences (Supplementary Data 2).

*ChIP-qRCR*

Chromatin was immunoprecipitated according to the user guide of the EZ‐Magna G Chromatin Immunoprecipitation kit (Millipore, Massachusetts, USA). Occupancy was assessed by quantitative PCR for samples precipitated with a specific antibody versus samples precipitated with the control immunoglobulin G (the antibodies used in ChIP are listed in Table S4) using the specific primers indicated in Table S 5.

*ChIP-seq*

ChIP sequencing (Chip-seq) and bioinformatics analysis were conducted by Shanghai GENEFUND according to their established procedures (Supplementary Data 3 and 4).

*IF*

Cells were cultured in chamber slides overnight and fixed with 3.7% formaldehyde in PBS for 20 min at room temperature, followed by permeabilization with 0.5% Triton X-100 in PBS for 20 min at room temperature. Cells were then blocked for nonspecific binding with 1% BSA in PBS for 1 h at room temperature and incubated with the C3b/RBBP4/Lamin A/C antibody overnight at 4 °C, followed by incubation with Alexa Fluor 488/555/647 goat anti-rabbit/mouse IgG (as shown in Table S4) for 2 h at room temperature. Cover slips were mounted on slides using antifade mounting medium with DAPI (DAPI Fluoromount-GTM, Yeasen Biotechnology, Shanghai, China). Immunofluorescence images were acquired with a confocal laser scanning microscope (Olympus Corporation, Tokyo, Japan).

*Gel chromatography*

Cell proteins were harvested in protein extraction buffer supplemented with protease inhibitors and phosphatase inhibitors (Beyotime Biotechnology, Shanghai, China) and then were filtrated by 0.22 μM filter. The gel chromatography column (Superdex 200; Amersham Biosciences, Marlborough, MA, USA) was washed and equilibrated with cold PBS (4 °C) before passing the extracts over the gel chromatography column. The flow rate was 0.3 mL·min−1. Fractions were collected at 0.3 mL per tube and analyzed by western blot. The molecular mass was determined by a Gel Chromatography Calibration Kit HMW (GE Healthcare, Marlborough, MA, USA).

**Table S1. siRNA/guide RNA used in this study**

| **Gene name** | **Sequences** |
| --- | --- |
| *siSIN3A-1* | sense: 5’-GGUGGAACAGAAUCGUUAUUU-3’  antisense: 5’-AAAUAACGAUUCUGUUCCACC-3’ |
| *siSIN3A-2* | sense: 5’-CUAGCACAGAAACCAGUAUUU-3’  antisense: 5’-AAAUACUGGUUUCUGUGCUAG-3’ |
| *siHDAC1/2-1* | sense: 5’-GAAGACACCUUGAUUAGAUUCUGUU-3’  antisense: 5’-AACAGAAUCUAAUCAAGGUGUCUUC-3’ |
| *siHDAC1/2-2* | sense: 5’-GAUAAAUCAGUAGCUUCCAGCUUCU-3’  antisense: 5’-AGAAGCUGGAAGCUACUGAUUUAUC-3’ |
| *Scrambled Control* | sense: 5’-UUCUCCGAACGUGUCACGUUU -3’  antisense: 5’-ACGUGACACGUUCGGAGAAUU-3’ |

**Table S2. Primers for PCR in this study**

| **Plasmids name** | **Sequences** |
| --- | --- |
| p-GEX-6P-1-HDAC1 | F: 5’-CGGGATCCATGGCGCAGACGCAGGGCAC-3’  R: 5’-TCCCCCGGGTCAGGCCAACTTGACCTCCT-3’ |
| p-GEX-6P-1-HDAC2 | F: 5’-CGGGATCCATGGCGTACAGTCAAGGAGG-3’  R: 5’-TCCCCCGGGTCAGGGGTTGCTGAGCTGTT-3’ |
| p-GEX-6P-1-SAP18 | F:5’-AGTTCTGTTCCAGGGGCCCATGCTCGCTGCAGG  GGTCG-3’  R: 5’-GCGGCCGCTCGAGTCGACCCTTAATATGGTCTC  ATGCGCCC -3’ |
| p-GEX-6P-1-ING2 | F: 5’-AAGTTCTGTTCCAGGGGCCCATGTTAGGGCAG  CAGCAG  R:5’CGGCCGCTCGAGTCGACCCCTACCTCGATCTTC  TATCCTTTTTT |
| pCDH-SIN3A | F: 5’-TTGACCTCCATAGAAGATTATGAAGCGGCGTT  TGGATGA-3’  R: 5’-GCGATCGCAGATCCTTCGCTTAAGGGGCTTTG  AATACTG-3’ |
| pCDH-GADD45A | F: 5’-GCTCTAGAATGACTTTGGAGGAATTCTCG-3’  R: 5’-ATAAGAATGCGGCCGCTCACCGTTCAGGGAG  ATTAA- 3’ |
| PCDNA3.1-C3-Flag-NLSm | 1F: 5’-GCTCTAGAATGGGACCCACCTCAGGTCC-3’  1R: GGTCTGCTGTTCGTCTTCGTCGGTGGTGGCC  AGGCTGCAGAAGGCTGGA-3’  2F: 5’-GCCACCACCGACGAAGACGAACAGCAGA  CCGTAACCATCCCCCCCAAGTC-3’  2R: 5’-CCCAAGCTTTCACTTGTCATCGTCGTCCTT  GTAATCGTTGGGGCACCCAAAGA-3’ |

**Table S3. Sequences of shRNA used in this study**

| **Gene name** | **Sequences** |
| --- | --- |
| sh*C3*-1 | 5’-CCGGGCCGGAAGGAATCAGAATGAAC  TCGAGTTCATTCTGATTCCTTCCGGCTTTTTG-3’  5’-AATTCAAAAGCCGGAAGGAATCAGAATGAA  CTCGAGTTCATTCTGATTCCTTCCGGC -3’ |
| sh*C3*-2 | F-5’- CCGGTGCCCAGTTTCGAGGTCATAGCTCGAG  CTATGACCTCGAAACTGGGCATTTTTTG -3’  R-5’-AATTCAAAAATGCCCAGTTTCGAGGTCATAG  CTCGAGCTATGACCTCGAAACTGGGCAT-3’ |
| sh*GADD45A*-1 | 5’-CCGGGAAGACCGAAAGGATGGATAACTCGAGTT  ATCCATCCTTTCGGTCTTCTTTTTG-3’  5’-AATTCAAAAAGAAGACCGAAAGGATGGATAACT  CGAGTTATCCATCCTTTCGGTCTTC-3’ |
| sh*GADD45A*-2 | 5’-CCGGGGATCCTGCCTTAAGTCAACTTATTCTCGA  GAATAAGTTGACTTAAGGCAGGATCCTTTTTG-3’  5’-AATTCAAAAAGGATCCTGCCTTAAGTCAACTT  ATTCTCGAGAATAAGTTGACTTAAGGCAGGATCC-3’ |
| sh*SIN3*A | 5’- CCGGGGTGGAACAGAATCGTTATTTCTCGAGAA  ATAACGATTCTGTTCCACCTTTTTG-3’  5’-AATTCAAAAAGGTGGAACAGAATCGTTATTTCTCG  AGAAATAACGATTCTGTTCCACC-3’ |
| shNC | 5’-CCGGAATTCTCCGAACGTGTCACGTCTCGAGACGTG  ACACGTTCGGAGAATTTTTTTG-3’  5’-AATTCAAAAAAATTCTCCGAACGTGTCACGTCTCG  AGACGTGACACGTTCGGAGAATT-3’ |
| *CTSL g-1*  *CTSL g-2*  *CTSL g-3* | sense: CACCGCTTTGTGGACATCCCTAAGC  antisense: AAACGCTTAGGGATGTCCACAAAGC  sense: CACCGCTTTCAAAACCGTAAGCCC  antisense: AAACGGGCTTACGGTTTTGAAAGC  sense: CACCGAGATGTTCCGGAAAACTGGG  antisense: AAACCCCAGTTTTCCGGAACATCTC |

**Table S4. Antibodies used in this study**

| **Antibodies** | **Manufacturers** | **Applications** | **Catalog Number** |
| --- | --- | --- | --- |
| C3/C3b | PROTEINTECH | IP(3μg/test)  WB（1：1,000）  ChIP (5μg/test) | 21337-1-AP |
| C3/C3b | Abcam | IF（1：100） | ab181147 |
| C3aR | Biolegend | FCM | 345804 |
| SIN3A | Abcam | IP (3μg/test)  WB (1:1,000)  ChIP (5μg/test) | ab3479 |
| HDAC1 | CST | ChIP (10μl/test)  WB (1:1,000) | 34589 |
| HDAC1 | PROTEINTECH | WB (1:1,000) | 66085-1-lg |
| HDAC2 | PROTEINTECH | WB (1:1,000) | 67165-1-lg |
| RBBP4 | PROTEINTECH | WB (1:1,000) | 66060-1-lg |
| RBBP7 | HuaBio | WB (1:1,000) | EM1712-17 |
| RBBP7 | PROTEINTECH | IP (3μg/test) | 20365-1-AP |
| SAP18 | PROTEINTECH | WB (1:1,000) | 13841-1-AP |
| SAP30 | PROTEINTECH | WB (1:1,000) | 27679-1-AP |
| ING2 | PROTEINTECH | WB (1:1,000) | 11560-1-AP |
| EZH2 | CST | WB (1:1,000) | 5246 |
| SUZ12 | PROTEINTECH | WB (1:1,000) | 20366-1-AP |
| MTA1 | CST | WB (1:1,000) | 5646 |
| CHD4 | PROTEINTECH | WB (1:1,000) | 14173-1-AP |
| Isotype IgG | ABclonal | IP (3μg/test)  ChIP (5μg/test) | AC005 |
| Isotype IgG | CST | IP (3μg/test) | 3900 |
| α-Tubulin | PROTEINTECH | WB (1:10,000) | HRP-66031 |
| Histone 3 | Abcam | WB (1:1,000) | ab1791 |
| β-Actin (C4) | Santa Cruz Biot | WB (1:500) | sc-47778 |
| DDDDK-tag | MBL | WB (1:1,000)  IP (3μg/test)  ICC (1:500) | M185 |
| GST (26H1) | Cell Signaling Technology | WB (1:1,000) | 2624 |
| 6×His | Abcam | WB (1:1,000) | ab18184 |
| H3Ac | ACTIVE MOTIF | ChIP (5μg/test) | 39139 |
| CTSL | CST | WB (1:1,000) | 71298 |
| Secondary antibody | PROTEINTECH | WB (1: 10,000) | SA00001-1 |
| Secondary antibody | PROTEINTECH | WB (1: 10,000) | SA00001-2 |
| Secondary antibody | PROTEINTECH | WB (1: 10,000) | SA00001-7L |
| Secondary antibody | Invitrogen | IF (1:500) | A-11029 |
| Secondary antibody | Invitrogen | IF (1:500) | A-21428 |
| Secondary antibody | Invitrogen | IF (1:500) | A-21235 |

**Table S5. Primers for qRT-PCR/Chip-QPCR in this study**

| **Gene name** | **Sequences** |
| --- | --- |
| *CDK1* | F: 5’-AAACTACAGGTCAAGTGGTAGCC-3’  R: 5’-TCCTGCATAAGCACATCCTGA-3’ |
| *CDC14B* | F: 5’-GCCATTCTCTACAGCAGACCA-3’  R: 5’-TGTAAACCATTGCCAGATTGAGT-3’ |
| *MSH2* | F: 5’-CACTGTCTGCGGTAATCAAGT-3’  R: 5’-CTCTGACTGCTGCAATATCCAAT -3’ |
| *XPC* | F: 5’-CATCGTGGGAGCCATCGTAAG-3’  R: 5’-CTCACCATCGCTGCACATTTT -3’ |
| *ING4* | F: 5’-TGCAAGGAATTTGGTGACGAC -3’  R: 5’-GCCGCCGAATGTGTTTGTC-3’ |
| *MRNIP* | F: 5’- TTGTGGAGAGAAGCAGTCCTT -3’  R: 5’- CCTTCACCATAAGCCTTTCCAGA - 3’ |
| *UIMC1* | F: 5’- TACCTACCCTAGCAGATGCCA-3’  R: 5’- GCAGAATGGAATACCCCAGAAA- 3’ |
| SDE2 | F: 5’- TGCACCGTCCGGGATTTTATC-3’  R: 5’-CTGCACTGTGTCACTGGTGTT-3’ |
| *GADD45A* | F: 5’-GAGAGCAGAAGACCGAAAGGA-3’  R: 5’-CAGTGATCGTGCGCTGACT-3’ |
| *BRCA1* | F: 5’-GAAACCGTGCCAAAAGACTTC-3’  R: 5’-CCAAGGTTAGAGAGTTGGACAC- 3’ |
| *MAD2L2* | F: 5’-TGGCTGTGCATCTCATCCTCT-3’  R: 5’-GCGGTGCTCTTTATCCAAAATCA-3’ |
| *CDKN1A* | F: 5’- TGTCCGTCAGAACCCATGC-3’  R: 5’-AAAGTCGAAGTTCCATCGCTC-3’ |
| *TRAP1* | F: 5’-AGGTGTTTATACGGGAGCTGA-3’  R: 5’-GCATTGGTCTGCAAGTGAATCTC-3’ |
| *SPRED1* | F: 5’-CAGCCAGGCTTGGACATTCA-3’  R: 5’-TGGGACTTTAGGCTTCCACAT- 3’ |
| *PIDD1* | F: 5’-TCAGAGGATTCGGACGCAG-3’  R: 5’-GTGAGTGCTCAGACGCAAGAA-3’ |
| *C3* | F: 5’-GGGGAGTCCCATGTACTCTATC-3’  R: 5’-GGAAGTCGTGGACAGTAACAG-3’ |
| *β-ACTIN* | F: 5’-CGGGAAATCGTGCGTGAC-3’  R: 5’-CAGGGAGGAGCTGGAAGC-3’ |
|  | **Primer sequences for ChIP-qPCR** |
| *GADD45A* | F: 5’-GACGAGGACGACGACAGAGAT-3’  R: 5’-CACGCAGTGCAGGTCCG-3’ |

**Supplementary Figures**

**Figure S1**


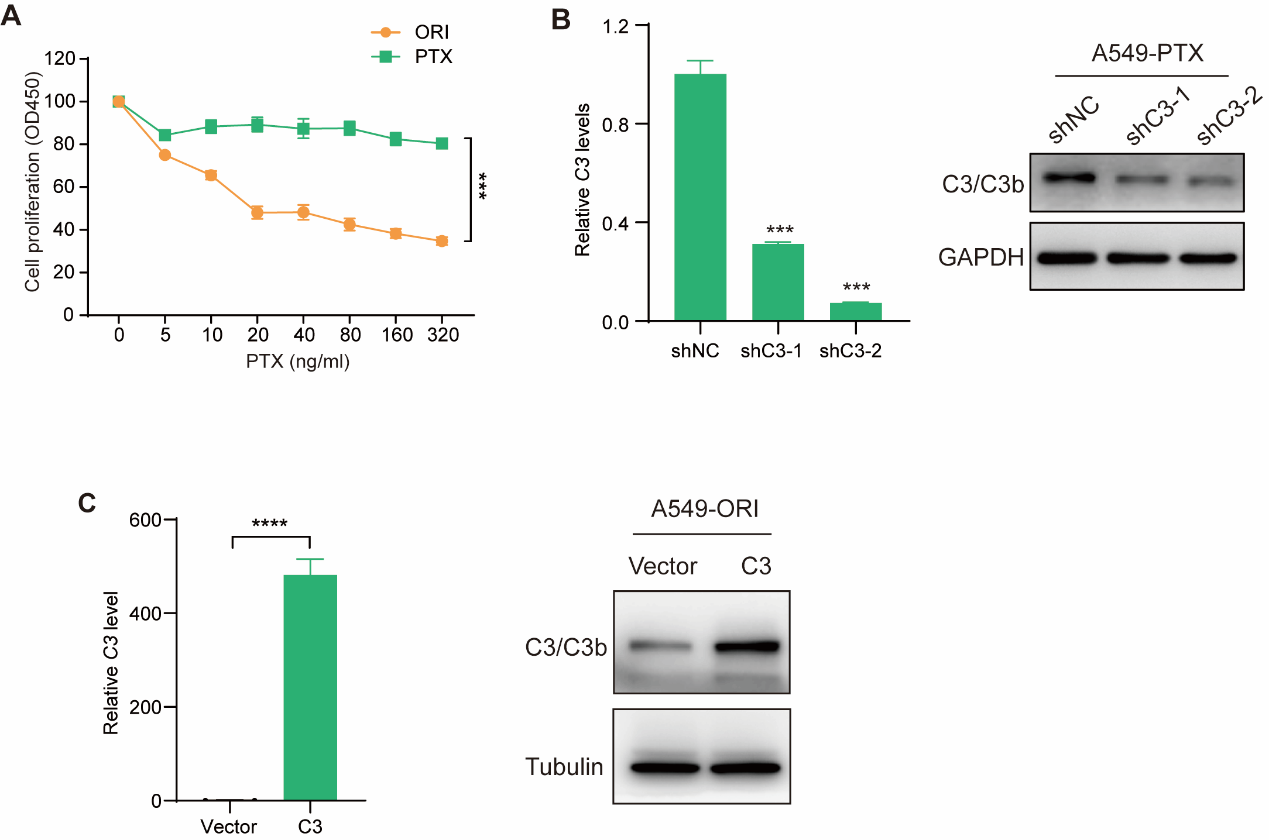


**Figure S1 The efficiency of overexpression or knockdown of C3 in A549-ORI or A549-PTX cells.**

A. Establishment of the A549-PTX-resistant cell line. Error bars represent the mean ± SD, n=5, two-tailed Student’s *t test*.

B. QPCR and immunoblotting measurement of the expression of C3 in A549-PTX-shNC, A549-PTX-shC3-1, and A549-PTX-shC3-2 cells. Error bars represent the mean ± SD, n=3, two-tailed Student’s *t test*.

C. QPCR and immunoblotting measurement of the expression of C3 in A549-ORI-vector and A549-ORI-C3 cells. Error bars represent the mean ± SD, n=3, two-tailed Student’s *t test*. **P*< 0.05, ***P* < 0.01, ****P*< 0.001 and *****P*< 0.0001.

**Figure S2**


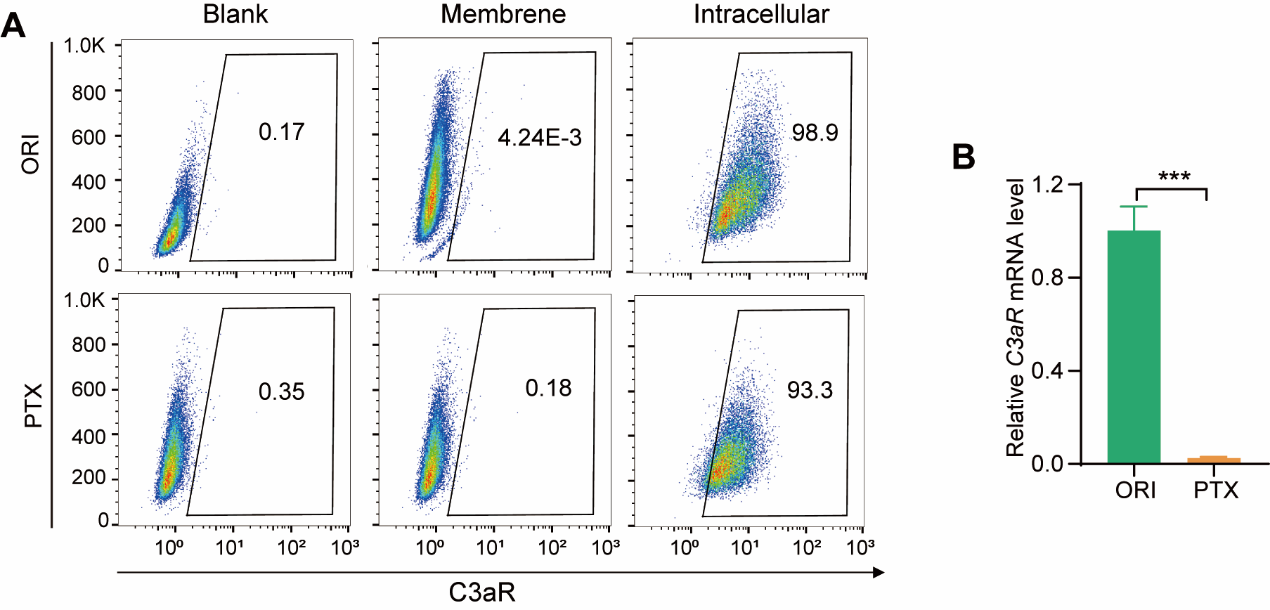


**Figure S2 The expression level of C3aR in A549-ORI or A549-PTX cells**

A. FCM analysis of C3aR in the cell membrane and cytoplasm in both A549-ORI and A549-PTX cells.

B. QPCR measurement of the expression of C3aR in A549-ORI and A549-PTX cells. Error bars represent the mean ± SD, n=3, two-tailed Student’s *t test*. **P*< 0.05, ***P* < 0.01, ****P*< 0.001 and *****P*< 0.0001.

**Figure S3**


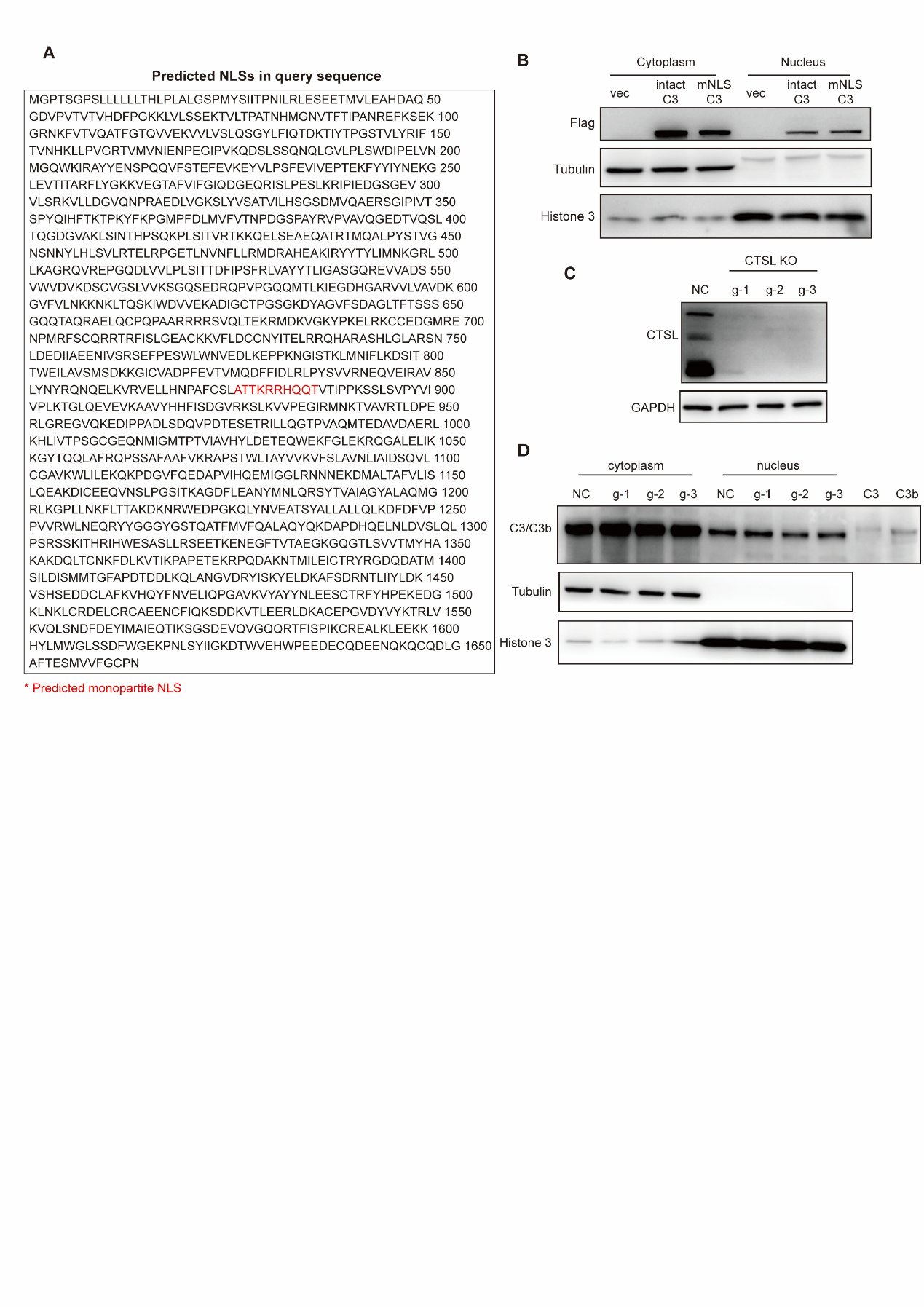


**Figure S3 The mechanism of C3/C3b entry into the nucleus in A549-PTX cells.**

A. The NLS sequence predicted by the NLS-mapper website.

B. Cytoplasmic and nuclear lysates of A549-vector, A549-C3 and A549-C3-NLSm cells were analyzed by IB with the indicated antibodies.

C. IB measurement of the expression of CTSL in scramble and CTSL knockout A549-PTX cells.

D. Cytoplasmic and nuclear lysates of A549-PTX-scramble and A549-PTX-CTSL-knockout cells by IB for C3 antibody.

**Figure S4**

**
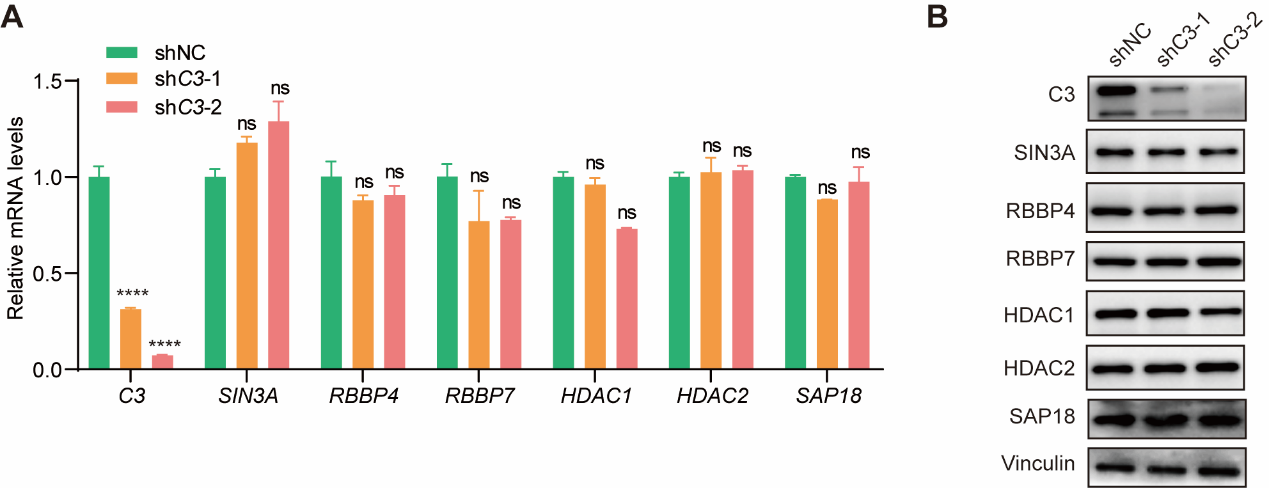
**

**Figure. S4 The expression levels of SIN3A complex components.**

A. QPCR measurement of the expression of the indicated genes in A549-PTX-shNC, A549-PTX-shC3-1, and A549-PTX-shC3-2 cells. Error bars represent the mean ± SD, n=3, two-tailed Student’s *t test*.

B. IB measurement of the protein levels of the indicated genes in A549-PTX-shNC, A549-PTX-shC3-1, and A549-PTX-shC3-2 cells. **P*< 0.05, ***P* < 0.01, ****P*< 0.001 and *****P*< 0.0001.

**Figure S5**

**
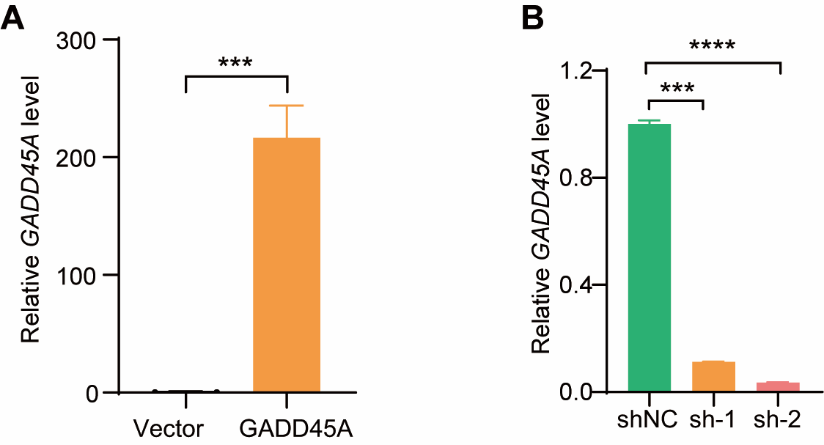
**

**Figure S5 The efficiency of overexpression or knockdown of GADD45A in A549-ORI or A549-PTX cells.**

A. QPCR measurement of the expression of GADD45A in A549-PTX-vector and A549-PTX-GADD45A cells. Error bars represent the mean ± SD, n=3, two-tailed Student’s *t test*.

B. QPCR measurement of the expression of GADD45A in A549-ORI-shNC, A549-ORI-shGADD45A-1, and A549-ORI-shGADD45A-2 cells. Error bars represent the mean ± SD, n=3, two-tailed Student’s *t test*. **P*< 0.05, ***P* < 0.01, ****P*< 0.001 and *****P*< 0.0001.

**Figure S6**

**
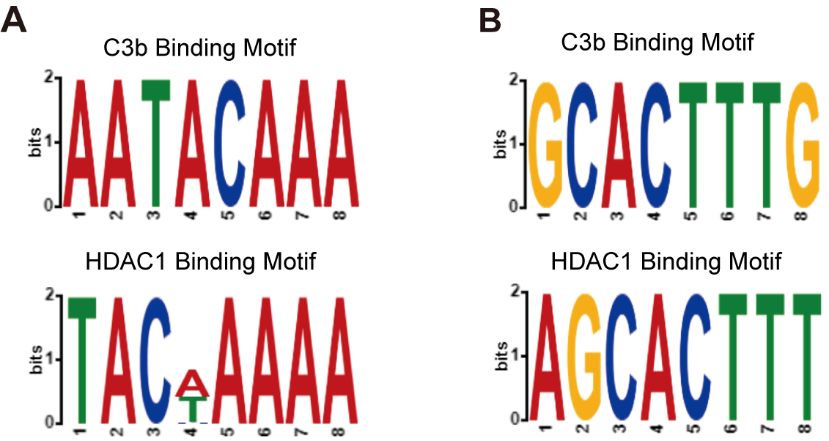
**

**Figure S6 DNA binding motifs of C3b and HDAC1 by MEME-ChIP analysis.**

A-B. DNA binding motifs of C3b and HDAC1 by MEME-ChIP analysis.
