# Supplementary material for: Nuclear complement C3b promotes paclitaxel resistance by assembling the SIN3A/HDAC1/2 complex in non-small cell lung cancer
Source: Cell Death Dis. 2023 Jun 8;14(6):351. doi: 10.1038/s41419-023-05869-y (PMC10250389; doi:10.1038/s41419-023-05869-y)
Supplement: Supplementary file 3 — Original Western Blot [file 41419_2023_5869_MOESM3_ESM.pdf]

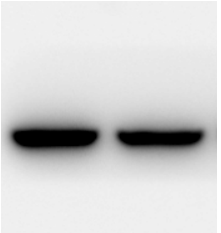

Figure 1-D-Actin

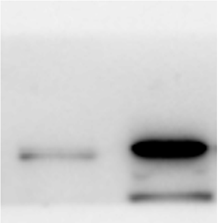

Figure 1-D-C3/C3b

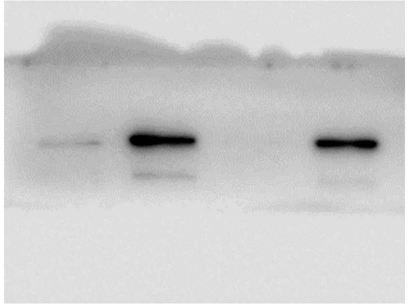

Figure 3-A-C3/C3b

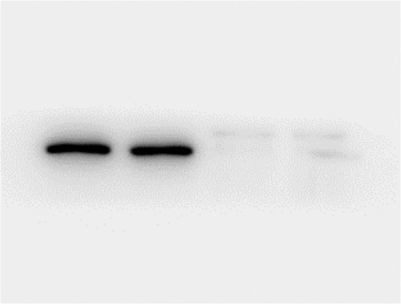

Figure 3-A-Tubulin

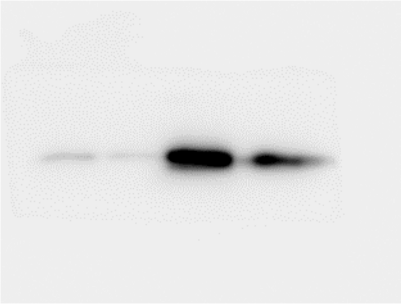

Figure 3-A-Histone H3

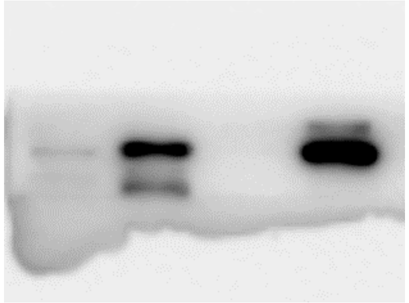

Figure 3-C-C3/C3b

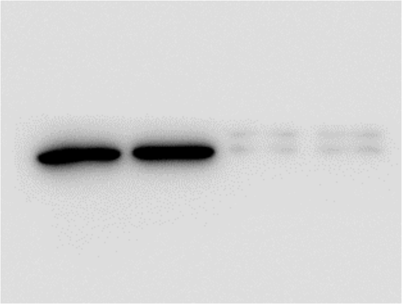

Figure 3-C-C3-Tubulin

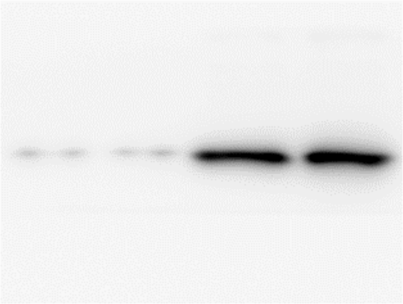

Figure 3-C-Histone H3

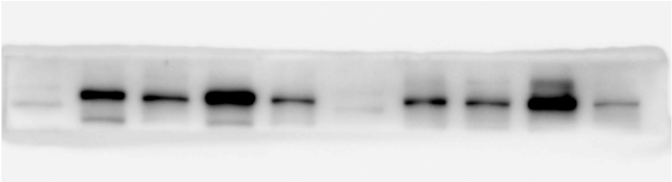

Figure 3-E-C3/C3b

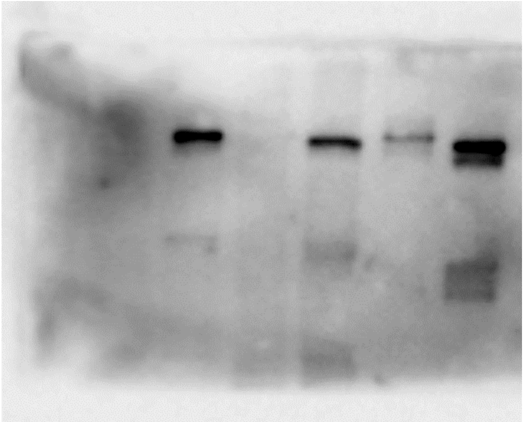

Figure 3-F-C3/C3b(Non-reduced Sample)

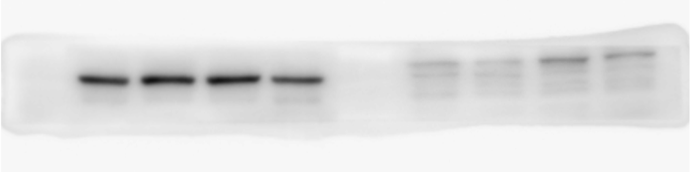

Figure 3-E-Tubulin

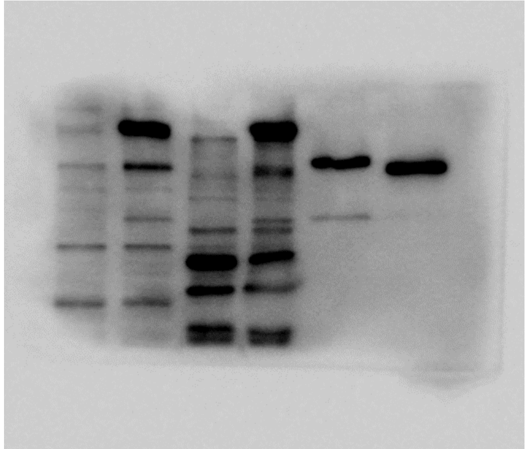

Figure 3-F-C3/C3b(Reduced Sample)

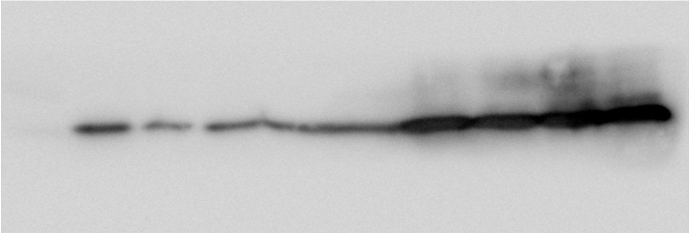

Figure 3-E-Histone 3

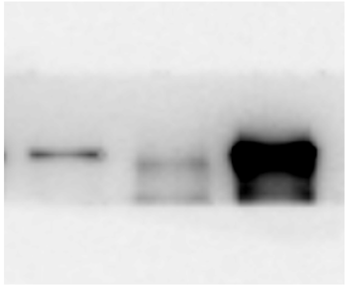

Figure 4-C-C3b

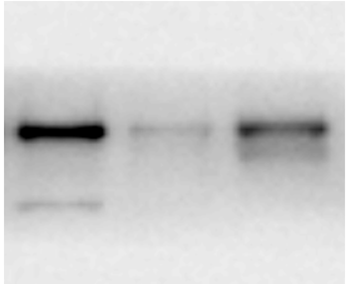

Figure 4-C-RBBP4

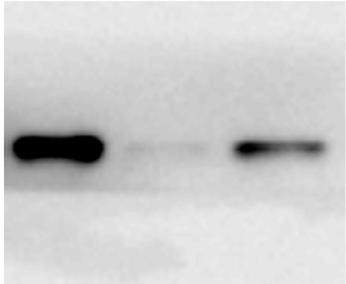

Figure 4-C-RBBP7

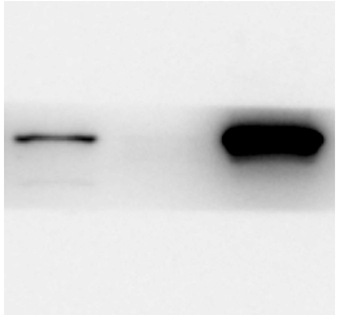

Figure 4-D-left-RBBP4

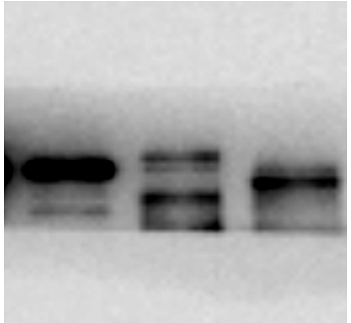

Figure 4-D-left-C3

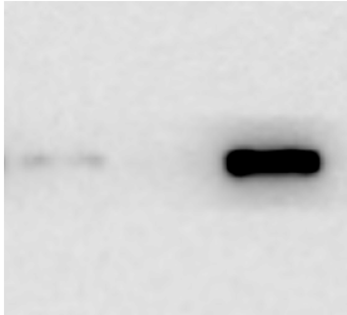

Figure 4-D-left-RBBP7

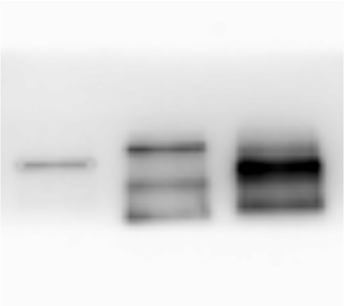

Figure 4-D-right-C3

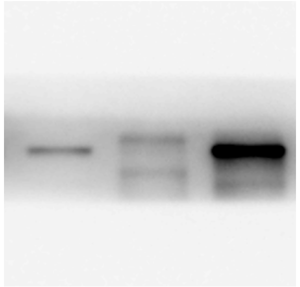

Figure 4-F-C3b

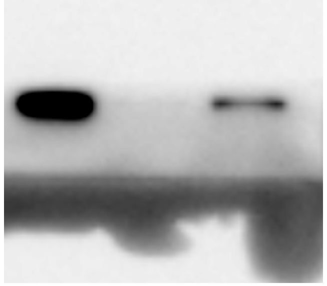

Figure 4-F-HDAC2

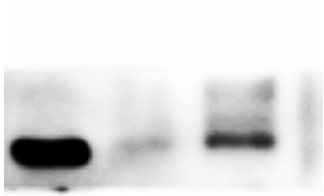

Figure 4-F-SAP30

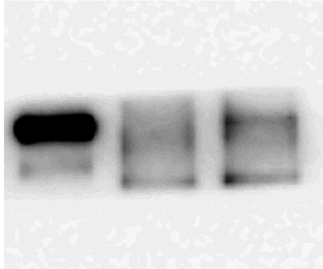

Figure 4-F-EZH2

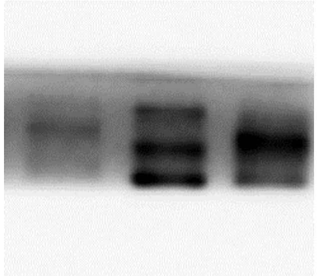

Figure 4-F-SIN3A

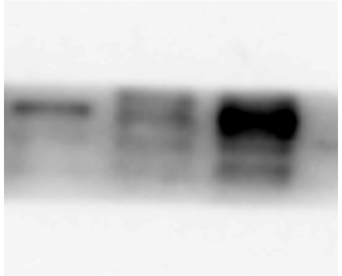

Figure 4-F-ING2

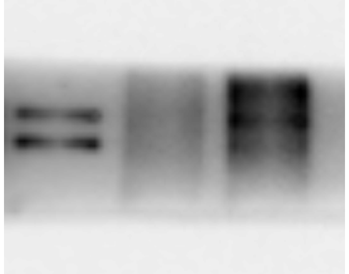

Figure 4-F-MTA1

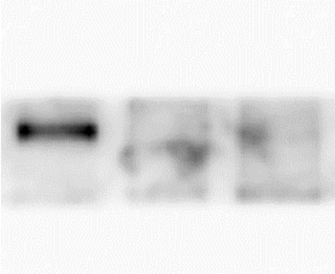

Figure 4-F-SUZ12

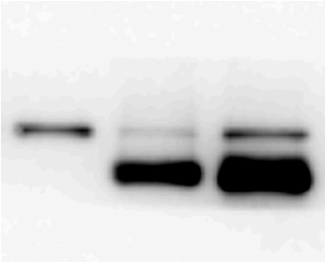

Figure 4-F-HDAC1

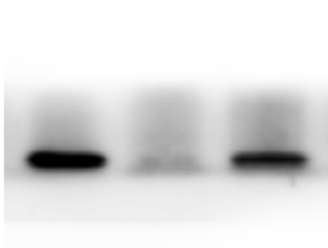

Figure 4-F-SAP18

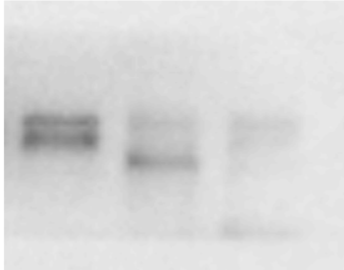

Figure 4-F-CHD4

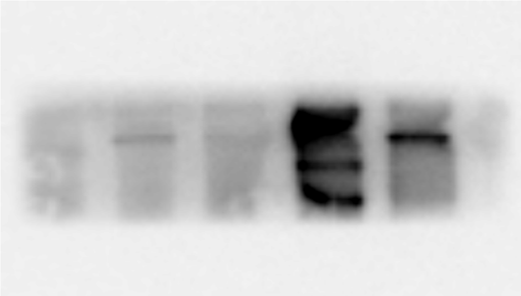

Figure 4-G-C3b

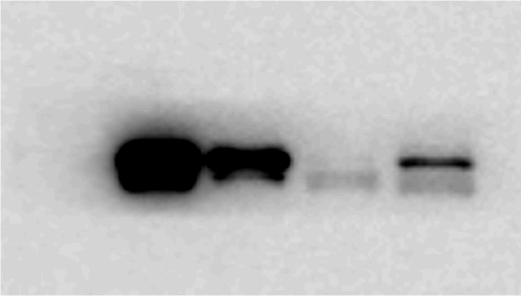

Figure 4-G-HDAC2

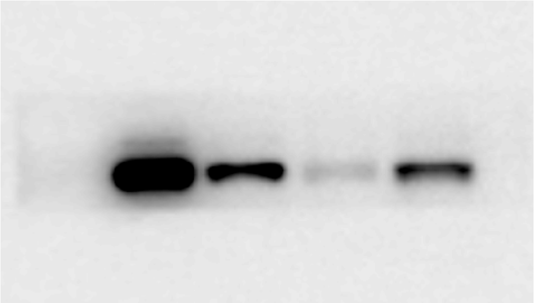

Figure 4-G-RBBP7

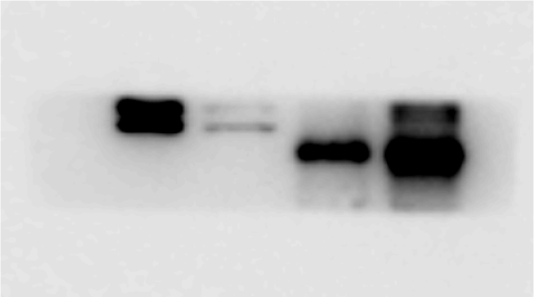

Figure 4-G-ING2

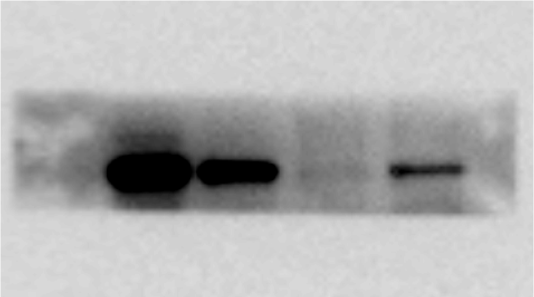

Figure 4-G-RBBP4

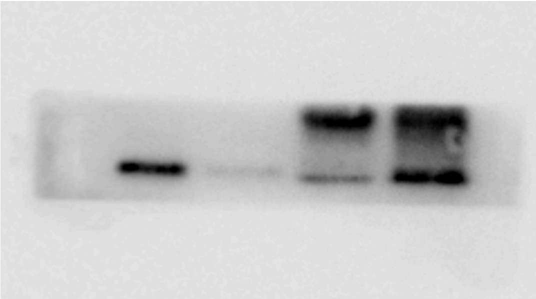

Figure 4-G-SAP18

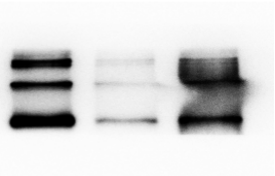

Figure 4-H-C3b

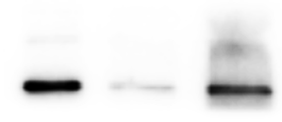

Figure 4-H-C3b

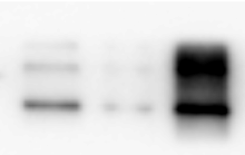

Figure 4-H-C3b

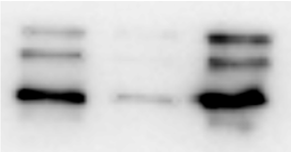

Figure 4-H-C3b

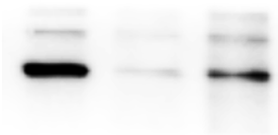

Figure 4-H-C3b

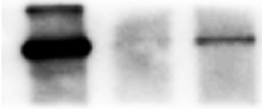

Figure 4-H-C3b

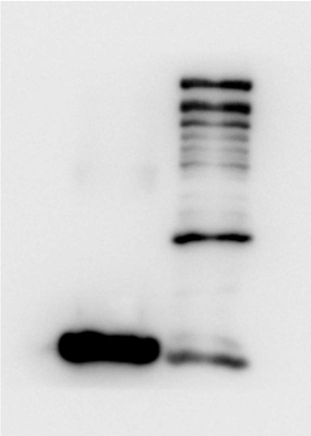

Figure 4-H-GST-HDAC1

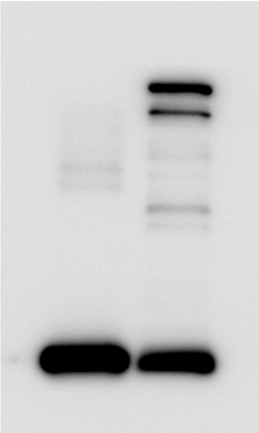

Figure 4-H-GST-HDAC2

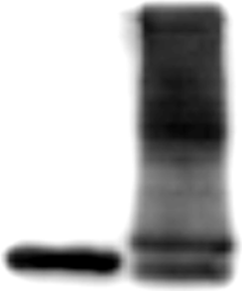

Figure 4-H-GST-RBBP4

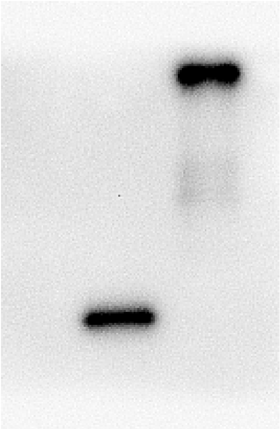

Figure 4-H-His-Sumo-RBBP7

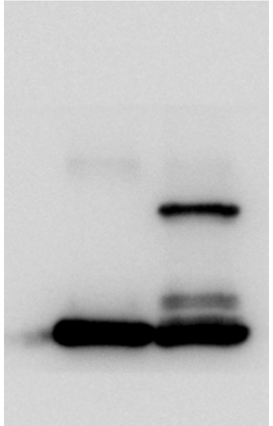

Figure 4-H-GST-SAP18

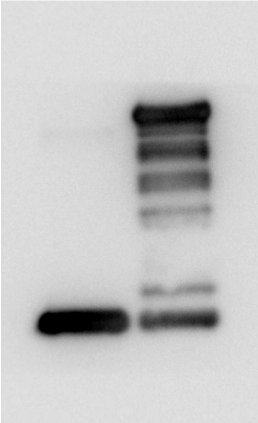

Figure 4-H-GST-ING2

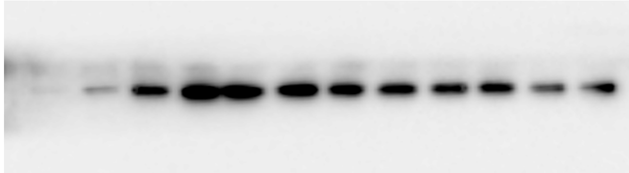

Figure 4-I-C3b

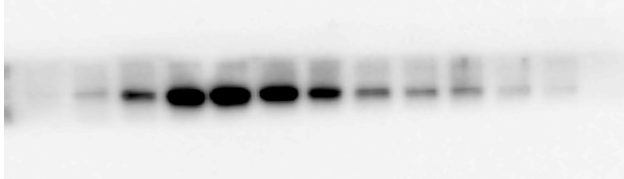

Figure 4-I-SIN3A

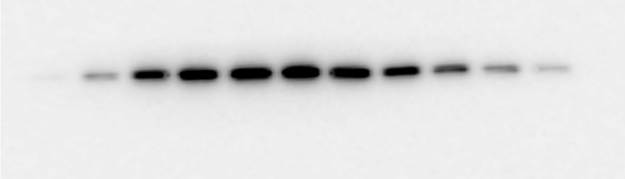

Figure 4-I-HDAC1

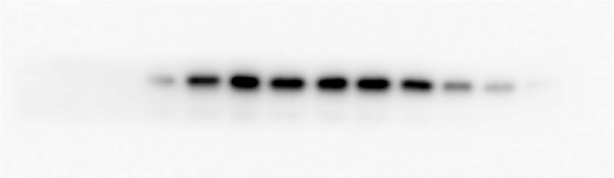

Figure 4-I-RBBP7

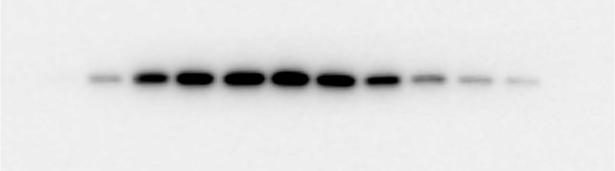

Figure 4-I-RBBP4

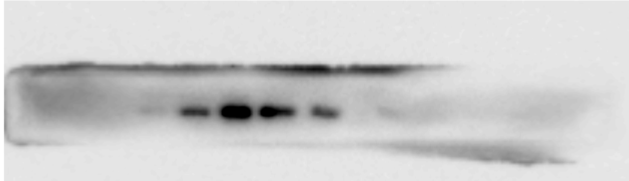

Figure 4-I-HDAC2

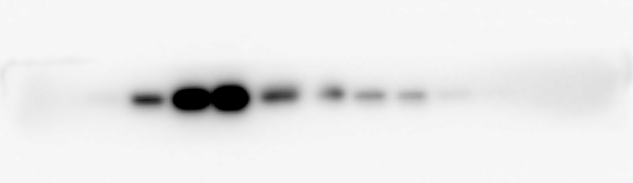

Figure 4-I-ING2

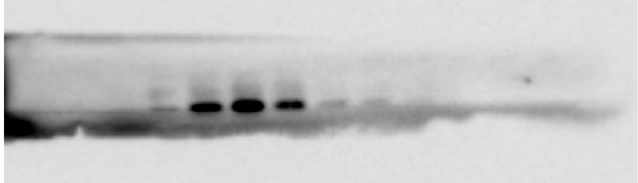

Figure 4-I-SAP30

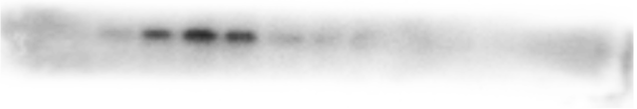

Figure 4-I-SAP18

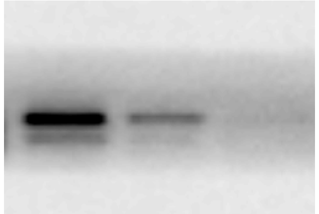
Figure 5-E-C3

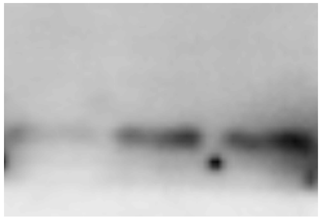
Figure 5-E-GADD45A

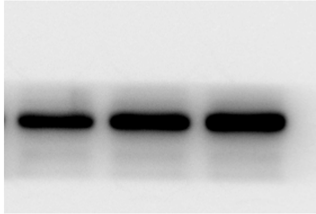
Figure 5-E-Tubulin

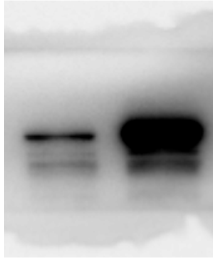
Figure 5-F-C3

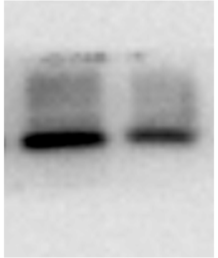
Figure 5-F-GADD45A

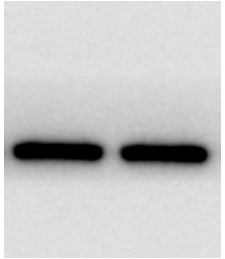
Figure 5-F-Tubulin

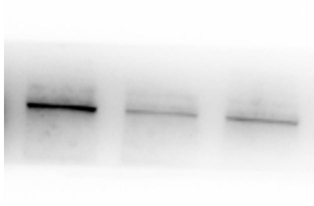

Figure 6-E-SIN3A

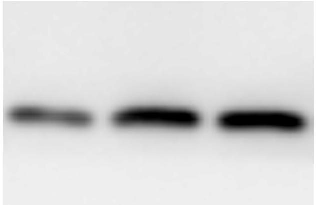

Figure 6-E-GADD45A

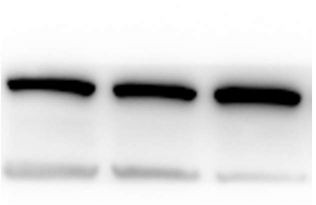

Figure 6-E-Tubulin

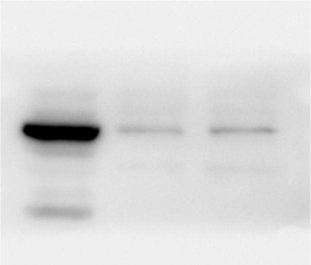

Figure 6-F-HDAC1

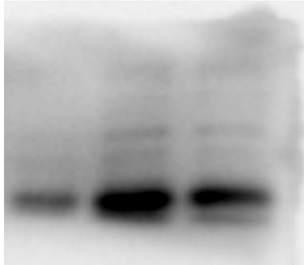

Figure 6-F-GADD45A

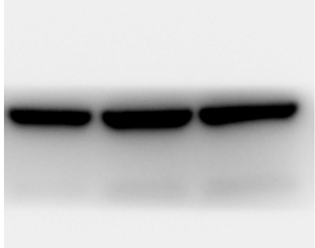

Figure 6-F-Actin

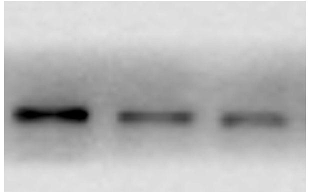

Figure S1-B-C3/C3b

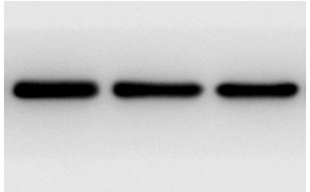

Figure S1-B-GAPDH

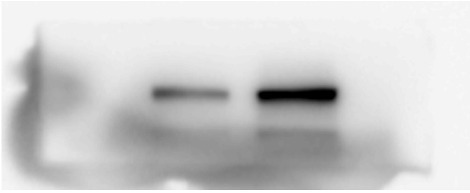

Figure S1-C-C3/C3b

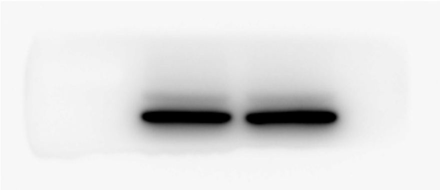

Figure S1-C-Tubulin

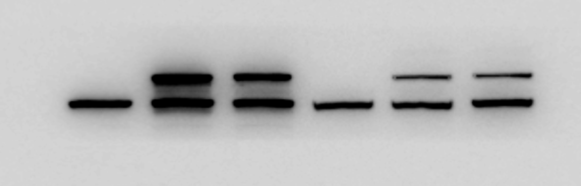

Figure S3-B-Flag

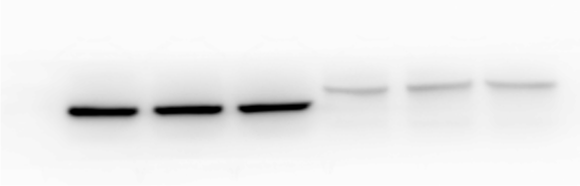

Figure S3-B-Tubulin

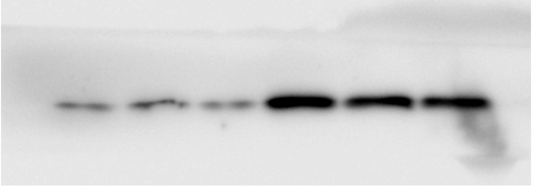

Figure S3-B-Histone 3

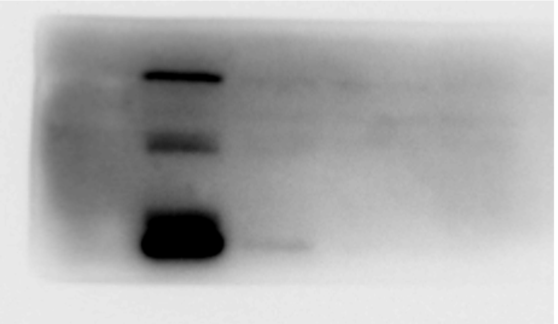

Figure S3-C-CTSL

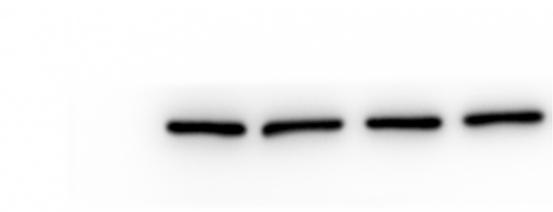

Figure S3-C-GAPDH

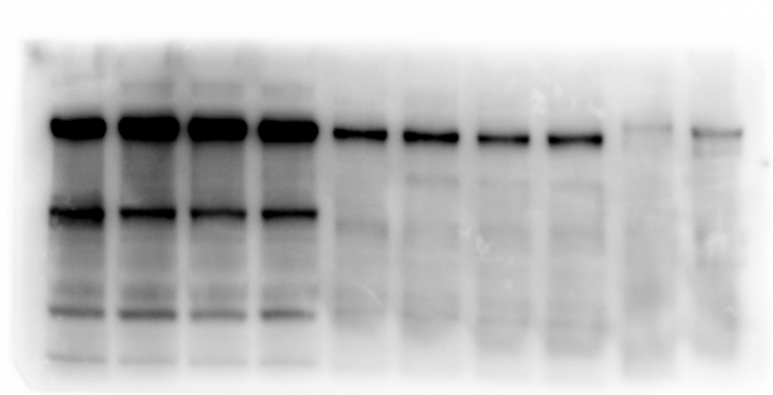

Figure S3-D-C3/C3b

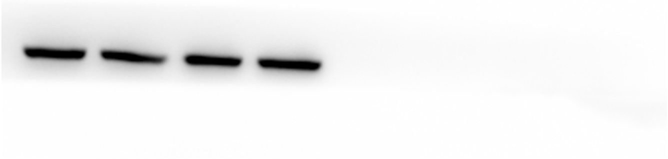

Figure S3-D-Tubulin

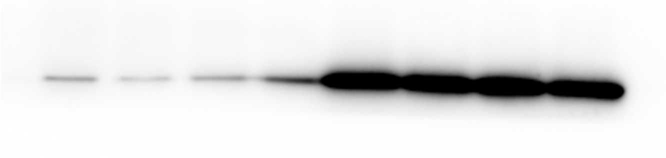

Figure S3-D-Histone 3

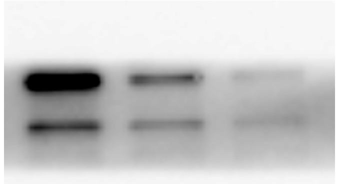

Figure S4-B-C3/C3b

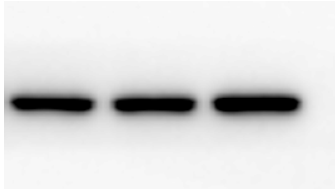

Figure S4-B-RBBP4

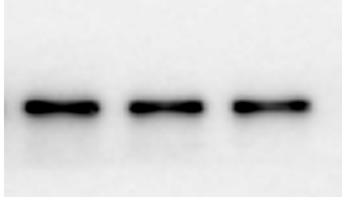

Figure S4-B-SIN3A

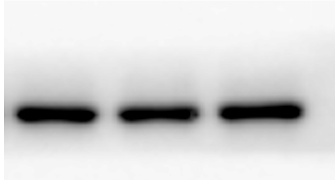

Figure S4-B-RBBP7

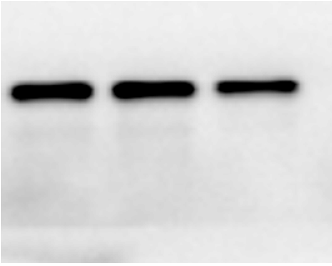

Figure S4-B-HDAC1

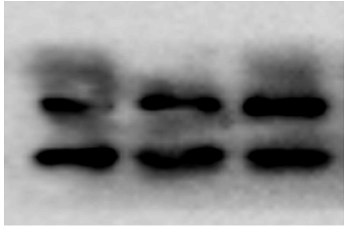

Figure S4-B-SAP18

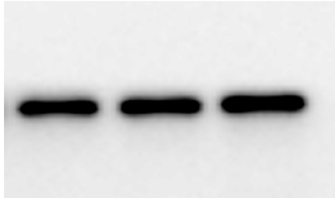

Figure S4-B-HDAC2

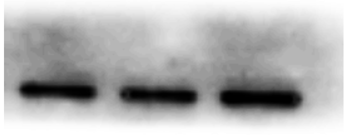

Figure S4-B-Vinculin
